# Phenotype and Response to PAMPs of Human Monocyte-Derived Foam Cells Obtained by Long-Term Culture in the Presence of oxLDLs

**DOI:** 10.3389/fimmu.2020.01592

**Published:** 2020-08-04

**Authors:** Anna Nogieć, Małgorzata Bzowska, Agnieszka Demczuk, Chen Varol, Krzysztof Guzik

**Affiliations:** ^1^Department of Immunology, Faculty of Biochemistry, Biophysics, and Biotechnology, Jagiellonian University, Kraków, Poland; ^2^The Research Center for Digestive Tract & Liver Diseases, The Tel Aviv Souraski Medical Center, Tel Aviv, Israel

**Keywords:** oxLDL, foam cells, macrophages, PAMPs, cytokines, atherosclerosis, inflammasome, pyroptosis

## Abstract

Cholesterol-laden, foam macrophages constitute the most characteristic component of human atherosclerotic plaques. Persistent uptake of oxLDLs results in accumulation of lipid bodies inside the cells and determines their phenotype and subsequent functions. In this work, we describe the phenotype of human monocyte-derived foam cells obtained by differentiation in the constant presence of oxLDLs for 30 days (prolonged-hMDFCs). Although neither the total cellular nor the cell surface expression of Toll-like receptors (TLR) was regulated by oxLDLs, the prolonged-hMDFCs changed dramatically their responsiveness to TLR ligands and inactivated bacteria. Using multiplex technology, we observed an acute decline in cytokine and chemokine production after surface and endosomal TLR stimulation with the exception of TLR2/6 triggering with agonists Pam2CSK4 and MALP-2. We also noted significant reduction of some surface receptors which can have accessory function in recognition of particulate antigens (CD47, CD81, and CD11b). In contrast, the prolonged-hMDFCs responded to inflammasome activation by LPS/nigericin with extensive, necrotic type cell death, which was partially independent of caspase-1. This pyroptosis-like cell death was aggravated by necrostatin-1 and rapamycin. These findings identify a potential contribution of mature foam cells to inflammatory status by increasing the immunogenic cell death burden. The observed cross-talk between foam cell death pathways may lead to recognition of a potential new marker for atherosclerosis disease severity. Overall, our study demonstrates that, in contrast to other cellular models of foam cells, the prolonged-hMDFCs acquire a functional phenotype which may help understanding the role of foam cells in the pathogenesis of atherosclerosis.

**Graphical Abstract:**
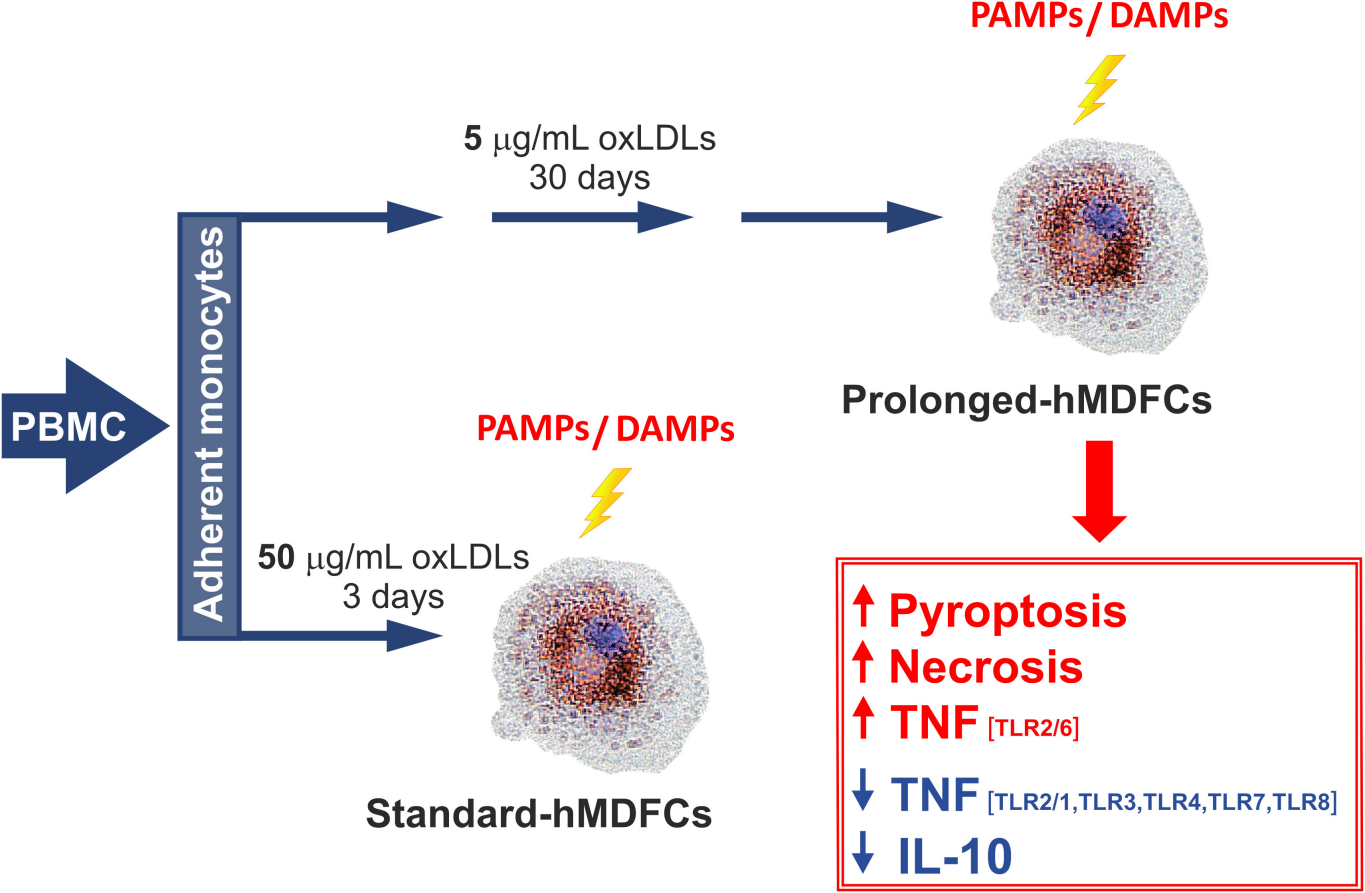


## Introduction

Atherosclerosis is clinically manifested as cardiovascular diseases, which are the number 1 cause of death globally (1). Although clinically relevant lesions become evident in middle-aged adults, it has been demonstrated that fat accumulation (known as fatty streaks) begins in early childhood (2). The latency period is long, and clinical manifestations become evident in middle-aged and older adults (3).

The hallmark of atherosclerosis is the low-grade, chronic inflammation of the arterial wall. One of the key processes responsible for atherosclerosis is the accumulation of monocyte/macrophage lineage cells within the lipid-rich subendothelial space of the affected artery (4). Consecutive formation of foam macrophages is a pivotal process in the development of the atherosclerotic plaque (5). Lipid-rich macrophages outnumber other cells in early plaques and are the main source of modulatory proteins present in the atheroma (6). Whereas, foam cell formation is a beneficial process in the early atherosclerotic lesion, in advanced lesions the foam cells die easily and release their contents (damage-associated molecular patterns, DAMPs) to the extracellular space (7, 8).

Cells in the atherosclerotic lesions can die in several ways, and the inflammatory response to each form of cell death is highly variable. In contrast to the homeostatic apoptosis, highly pro-inflammatory necrotic types of cell death, such as necroptosis and pyroptosis tend to trigger an inflammatory response (9). Pyroptosis is a form of cell death associated with inflammasome activation and requires the activity of caspase-1 (10). Pyroptotic cell death is highly proinflammatory because it leads to the release of not only intracellular contents (DAMPs) but also cytokines, such as IL-1β and IL-18. Necroptosis, a programmed form of necrosis, does not involve caspase activation. Necroptosis leads to disruption of the plasma membrane and release of the cellular contents and various DAMPs. Atherosclerotic plaques are populated mostly by macrophages (11), which equipped with a set of pattern recognition receptors (PRRs), including Toll-like and scavenger receptors, readily respond to DAMPs. Several studies have shown that inflammation caused by TLR activation by endogenous ligands participates in the development of atherosclerosis (12–14).

Furthermore, microbial pathogens promote both the induction and perpetuation of atherosclerotic lesions. The involvement of pathogens in atherosclerosis initiation and progression is supported by multiple epidemiological surveys (15). Many studies have documented that the risk of developing atherosclerosis is correlated with chronic infections by various pathogens, including bacteria, such as *Chlamydia pneumoniae, Helicobacter pylori*, and *Porphyromonas gingivalis* as well as viruses, such as cytomegalovirus, HIV, and influenza A virus (16). Lesional macrophages respond to pathogens and pathogen associated molecular patterns (PAMPs) using the same PRRs which serve to recognize endogenous danger signals—it worsens the DAMPs-induced inflammatory status and undermines the structural stability of the plaque (16). Thus, bacteria and bacterial structures present in atheroma contribute to enhanced risk of plaque rupture (17). Apart from danger signals, macrophages present in the local microenvironment of the atherosclerotic intima are exposed to growth factors, cytokines, specialized pro-resolving and pro-inflammatory mediators (leukotrienes), and many lipids and lipid-containing molecules (5, 18).

Because infiltration and accumulation of the atherogenic plasma lipoproteins is the driving force of atherogenesis, removal of these lipoproteins in the developing lesions is the primary function of the intimal macrophages. Initially, the internalization and degradation of subendothelially retained lipoproteins by intimal macrophages can represent a process that delays lesion progression. Formation of foam cell is mainly due to uncontrolled uptake of modified low-density lipoprotein (LDLs) or impaired cholesterol efflux in macrophages, resulting in an excessive level of lipoprotein-derived cholesterol, which is consequently processed and accumulated inside the cells (19). These modified LDLs operate as *de facto* damage-associated molecular patterns, and are therefore recognized by host pattern recognition receptors, similar to how PAMPs are recognized (20).

The complex plaque microenvironement determines polarization of macrophages residing in the atheroma. In return, different phenotypes of macrophages shape plaque ecosystem, evolution and stability (5). Given the complexity of the plaque microenvironment, lesional macrophages are unlikely to exist in their pure M1/M2 polarized forms and instead represent intermediate states in a whole spectrum of phenotypes (7, 21).

Chronic (long-term) accumulation of foam cells in the intima takes place in the constant presence of proatherogenic lipids—oxidatively modified low density lipoproteins (oxLDLs). Most cellular models of foam macrophages include stimulation with oxLDLs in relatively high concentrations and subsequent culture for 3–6 days. As helpful these cellular models may be, they are oblivious to the cumulative effects of modified lipoproteins, to which macrophages are continuously exposed in atheroma, and which cannot be imitated by a single episode of intensive oxLDLs phagocytosis. The prolonged *in vitro* culture of foam macrophages in the presence of oxLDLs may also reflect some emerging concepts of foam cells: (i) although precise data are not available, the lifetime of macrophages in atheroma is long (months not days), both for the resident and monocyte-derived cells (22, 23), (ii) diet-induced macrophage reprogramming is a gradual process (24) and (iii) increased lipid uptake and full development of the foam phenotype apparently involves a multilayered positive feedback-loop with oxLDLs and CD36 (25).

The aim of the present study was to determine whether our modified cellular model−30 days of differentiation in the constant presence of oxLDLs—is more adequate to study the biology of foam cells. The results presented below reveal novel aspects of immune activity of foam cells, and propose a new *in vitro* model which may help to study elements of plaque homeostasis.

## Materials and Methods

### Culture of Human Monocyte-Derived Macrophages (hMDMs) and Foam Cells (hMDFCs)

**Figure 1:**
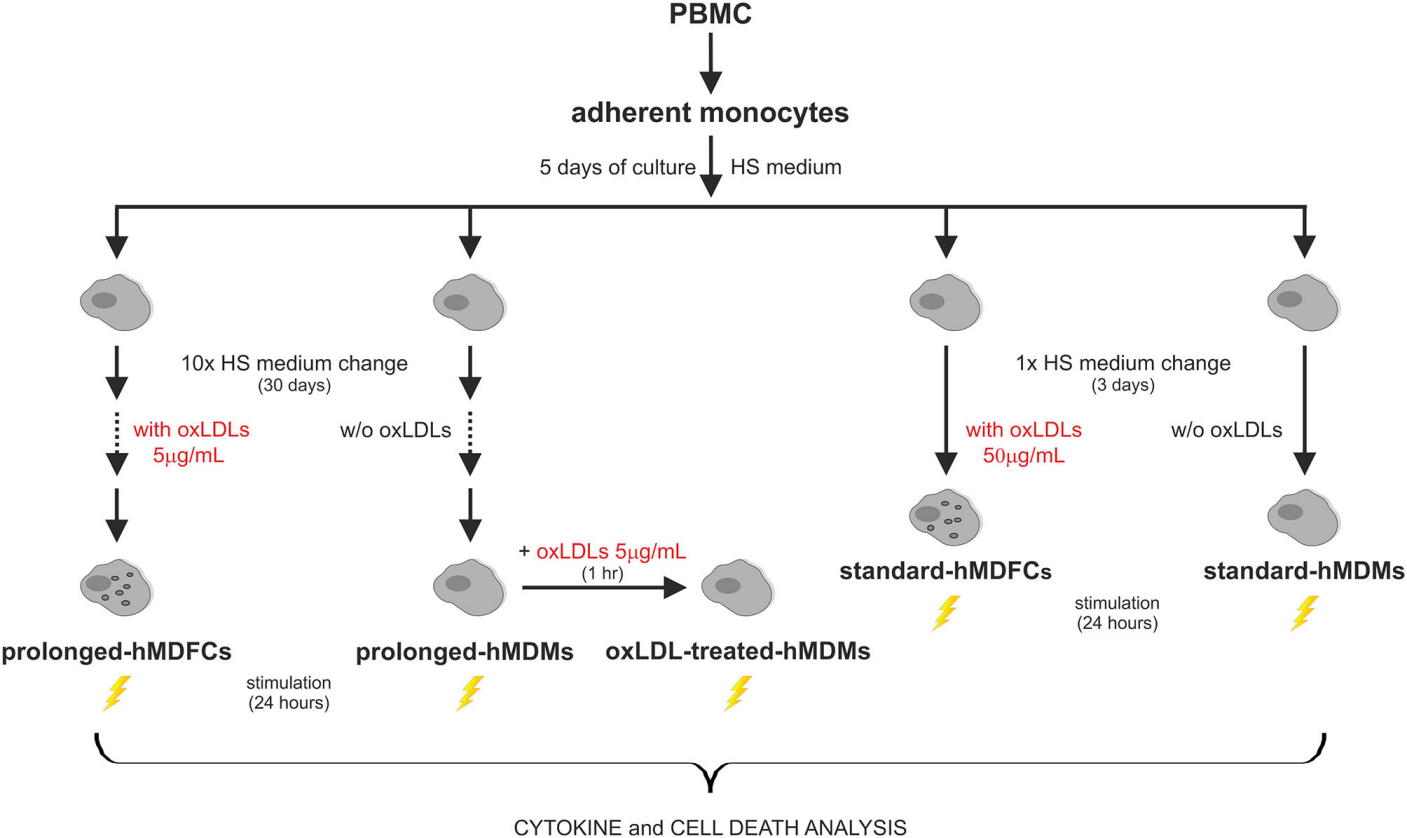
The outline of experimental protocol.

The outline of experimental protocol was shown in Figure 1. Peripheral blood mononuclear cells (PBMC) were isolated from citrate-treated blood of de-identified, healthy, normolipemic human donors obtained from the Regional Center for Blood Donation and Blood Treatment in Krakow (Poland) by standard density gradient separation (Pancoll human, PAN-biotech, Germany). The cells designed for cytokine analysis were plated at 4 × 10^6^ per well on 24-well plates (BD Primaria, BD Biosciences, USA) in RPMI1640 supplemented with L-glutamine (2 mM), gentamycin (50 μg/mL) and 10% fetal calf serum. After 2 h of incubation at 37°C in humidified atmosphere containing 5% CO_2_, the supernatant with non-adherent lymphocyte fraction was discarded. The remaining adherent monocytes were cultured in RPMI1640 supplemented with 12% heat-inactivated pooled human serum (HS medium) for 5 days to allow their differentiation to macrophages. Since the 5th day the cells were maintained either in HS medium (standard-hMDMs, prolonged-hMDMs, oxLDL-treated hMDMs), HS medium with addition of 50 μg/mL oxLDLs for next 3 days (standard-hMDFCs) or HS medium with addition of 5 μg/mL oxLDLs for a total time period of 30 days (prolonged-hMDFCs). The culture media were changed every 3 days. For cytometric measurements and immunoblotting PMBC were seeded at 20 × 10^6^ per well on 6-well culture plates (BD Primaria, BD Biosciences, USA) and treated as described above. Total serum cholesterol and triglycerides concentration in donors serum were determined with enzymatic diagnostic tests (LiquickCor-CHOL, LiquickCor-TG, CORMAY, Poland).

### Isolation and Oxidation of Low Density Lipoproteins

Low density lipoproteins (LDLs) were prepared from EDTA-treated plasma of healthy de-identified human donors obtained from the Regional Center for Blood Donation and Blood Treatment in Krakow (Poland). LDLs were isolated by sequential ultracentrifugation through a discontinuous KBr gradient according to Havel et al. (26). Plasma density was adjusted to 1.019 g/mL with solid KBr and centrifuged at 180,000 g for 24 h at 4°C (Beckman L7-65 ultracentrifuge with Ti 60 rotor, Beckman Coulter, Brea, USA). The top fraction of the gradient was gently removed. Density of the remaining solution was raised to 1.063 g/mL by addition of KBr and again centrifugated. LDLs fraction was collected and dialysed overnight at 4°C against PBS pH 7.4. Oxidation of LDLs was performed by incubation with 5 μM Cu^2+^ for 20 h at 37°C after adjustment of protein concentration to 0.12 mg/mL. Obtained oxLDLs were dialysed overnight against PBS pH 7.4 at 4°C. Preparations were concentrated by ultrafiltration (Amicon Ultra Centifugal Filters, 100K NMWL, Millipore, Ireland) at 3,500 g at 4°C and sterilized by filtration through a 0.22 μm syringe filter (Millex-GV, Millipore, Ireland). Protein concentration of the specimen was measured with Lowry method. The purity of preparations was controlled by polyacrylamide gel electrophoresis and subsequent gel staining with Oil Red O for lipids and with Coomasie Brilliant Blue R for proteins detection. All materials used during isolation procedures were endotoxin-free and we did not observe LDL- induced production of TNF and other markers of activation in human monocytes and macrophages. As a control of ultracentrifugation stages we used commercially available ultrapure LDL preparation (KALEN Human LDL™, KALEN Biomedical, LLC, USA) which was oxidized and treated as described above. We found no differences in activity between the two preparations.

### Spontaneous and PAMPs-Induced Protein Secretion Analysis

To analyze spontaneous and PAMPs-induced protein secretion, culture medium was replaced either with RPMI1640 with 2% HS (prolonged-hMDMs) or RPMI1640 with 2% HS additionally supplemented with 5 μg/mL of oxLDLs (prolonged-hMDFCs, oxLDL-treated hMDMs). The supernatants were collected after 24 h. For the stimulation with PAMPs, the culture media were changed 1 h before assay as described for spontaneous secretion and the cells were stimulated with indicated concentrations of PAMPs molecules or bacteria: stLPS and upLPS (10 ng/mL); MALP-2 (15 ng/mL) Pam2CSK4 and Pam3CSK4 (50 ng/mL), LTA and CL075 (1 μg/mL), Poly (I:C) (50 μg/mL). *P. gingivalis* were given at a 1:10 cell: bacterium ratio. Culture supernatants were collected 24 h after stimulation. To determine average protein content in the cultures, the cells were lysed with RIPA buffer containing protease inhibitors. Protein concentration were determined using micro Lowry method.

### Activation of NLRP3 Inflammasome

To analyse NLRP3 inflammasome activation, standard- and prolonged-hMDMs and hMDFCs were primed for 4 h with 1 μg/mL LPS and then treated with 10 μM nigericin for 20 h. For inhibition of caspase-3,−1,−8,−9; caspase-1 or RIP1 kinase activity, cells were treated with 20 μM Q-VD-OPh, 50 μM Ac-YVAD-cmk, or 20 μM necrostatin-1, respectively, for 1 h prior to LPS stimulation. For induction of autophagy, cells were treated with 50 nM rapamycin for 1 h before LPS stimulation. The supernatants were collected, centrifuged at 500 g for 5 min at 4°C and used immediately for lactate dehydrogenase (LDH) release assay and then frozen for cytokine measurement.

### Oil Red O Staining Procedure

Macrophages were gently washed with PBS, fixed with 4% phosphate buffered formalin for 15 min at 37°C and rinsed with PBS. The cells were then treated with 60% isopropanol for 15 s and stained in the darkness with Oil Red O solution for 30 min at 37°C. Oil Red O solution was freshly prepared by dilution of the stock solution (60 mg Oil Red O dissolved in 20 mL of 100% isopropanol) with distilled water in the ratio 3:2 and subsequent filtration. After washing with PBS the cells were counterstained with hematoxylin for 2 min, rinsed with PBS and analyzed under Nikon Eclipse Ti (Nikon, USA) microscope.

### Flow Cytometry

Macrophages were harvested, pelleted and resuspended in PBS containing 5% FCS (1.5 × 10^5^ cells/100 μl). The cells were stained with fluorescent-labeled anti-human antibodies or appropriate isotype controls for 30 min in the dark at 4°C. Because of elevated autofluorescence exhibited by hMDFCs, we employed quenching method which was described previously for analysis of alveolar macrophages and immature dendritic cells (27, 28). Accordingly, after washing with 1% FCS in PBS the cells were incubated with ice-cold 0.4% crystal violet solution in PBS pH 7.4 for 5 min. This step was omitted if APC-conjugated antibodies were used. Prior to analysis, the macrophages were washed extensively and suspended in 5% FBS in PBS. To determine the positive population an Overtone subtraction algorithm was applied. To compare the surface expression level of the markers expressed by the majority (over 60%) of the cells, mean fluorescence intensity (MFI) was used. Determination of MFI from 10,000 gated events from each sample was accomplished using LSRII cytometer (Becton Dickinson, USA) and BD FACSDiva™ software. Overtone subtraction statistical analysis and histograms overlays were performed using FCS Express™ (De Novo Software, USA) and FlowJo™ (FlowJO, LLC, Data Analysis Software, USA), respectively.

### Western Blot Analysis

Macrophages were lysed on plates with RIPA buffer (Sigma Aldrich, USA) containing protease inhibitors. Lysates were cleared by centrifugation at 8,000 g for 10 min at 4°C. Protein content was determined using microLowry method. Lysates were electrophoresed and immunobloted using standard conditions. Briefly, denatured protein samples (20–40 μg) were run on 4–12% SDS-PAGE and then transferred to a PVDF membrane (Amersham Biosciences Corp., UK). Membranes were blocked in 5% non-fat dry milk (BioShop, Canada) for 2 h followed by washing and overnight incubation with specific monoclonal (anti-CD11b, CD47, CD81) or polyclonal (anti- CD36, β-actin, IL-1β, GAPDH) antibodies. The bands were visualized with HRP-conjugated goat anti-rabbit, goat anti-mouse, or rabbit anti-goat antibodies. The chemiluminescence signal was detected and analyzed with ChemiDoc™ XRS+ System with Image Lab™ Software (Bio-Rad, USA).

### Luminex®-Based and ELISA Analysis of Cytokines and Chemokines

The analysis of the cytokines and chemokines secreted by macrophages were performed with FlexMAP 3D (Luminex®) platform using the Human Cytokine Magnetic 30-Plex Panel kit (Invitrogen, USA). The detection thresholds (in pg/mL) for particular protein were as shown in Table 1. The procedure was performed according to manufacturer instructions. All data were normalized to total protein concentration in the cell cultures. To confirm the results obtained by multiplexed bead-based immunoassay we applied sandwich ELISA Sets (BD OptEIA™ (BD Biosciences, USA) for human IL-6, IL-10 and TNF receiving consistent results. In order to determine IL-1β concentrations in cell culture supernatants upon NLRP3 inflammasome activation, Human IL-1β ELISA Set II was used according to the manufacturer's instructions. The sensitivity of the test was about 3.9 pg/mL.

**Table 1:** Proteins analyzed by multiplex assay and their detection thresholds.

**Analyte**	**Detection threshold (pg/ml)**
IL-1β	25
IL-2	10
IL-4	5
IL-5	3
IL-6	3
IL-7	30
IL-8	3
IL-10	5
IL-12	15
IL-13	10
IL-15	125
IL-17	50
IFNα	25
IFNγ	5
TNF	10
IL-1RA	60
IL-2R	60
Eotaxin	5
IP-10	5
MCP-1	10
MIG	45
MIP-1alpha	16
MIP-1beta	100
RANTES	20
EGF	40
FGF basic	22
G-CSF	30
GM-CSF	15
HGF	50
VEGF	10

### Lactate Dehydrogenase (LDH) Assay

LDH release to the cell culture medium upon NLRP3 inflammasome activation was measured using Pierce™ LDH Cytotoxicity Assay Kit in accordance with the manufacturer's instructions. LDH release was normalized to total LDH content (cells treated with lysis buffer).

### Reagents and Antibodies

The heat-killed bacteria *Porphyromonas gingivalis* (PG) strain W83 was kindly provided by A. Sroka and purified *S. aureus* lipoteichoic acid (LTA) was kindly provided by Dr. J. Kozieł (both from Department of Microbiology, Faculty of Biochemistry, Biophysics and Biotechnology, Jagiellonian University, Poland). Ultrapure *E. coli* 011:B4 LPS (upLPS), Pam2CysSerLys4 (Pam2), Pam3CysSerLys4 (Pam3), CL075, Poly (I:C), selective caspase-1 inhibitor (Ac-YVAD-cmk), and rapamycin were purchased from Invivogen, USA. Macrophage-activating lipopeptide-2 (MALP-2) was obtained from Imgenex Corp., USA. *E. coli* 0127:B8 LPS (stLPS), nigericin, necrostatin-1, copper (II) sulfate, glutamine–gentamicine solution, hematoxylin, Oil Red O powder, crystal violet, potassium bromide, RIPA buffer and Total Protein Kit, MicroLowry, Petersons Modification were supplied by Sigma Aldrich, USA. Complete Protease Inhibitor Cocktail Tablets were purchased from Roche Diagnostics, Germany. Pan-caspase inhibitor (Q-VD-OPh) was from R&D Systems, USA. Fetal calf serum was obtained from Biochrom GmbH, Germany. RPMI 1640 and 10× Dulbecco's phosphate-buffered saline (PBS) was from Gibco by Life Technologies, UK.

Primary mouse antihuman antibodies used in this study include the following: FITC-conjugated anti-TLR6 (TLR6.127), CD11b (VIM12), PE-conjugated anti-CD14 (Tuk4) and rabbit anti-GAPDH from Abcam, UK; FITC-conjugated mouse anti-CD16 (3G8), PE-conjugated anti-CD18 (6.7), CD47 (B6412), CD81 (JS-81), CD91 (A2MRa2), DC-SIGN (DCN46), APC-conjugated anti-CD36 antibody obtained from BD Biosciences, USA; Alexa Fluor 488-conjugated anti-TLR2 (TL2.1), TLR4 (HTA125), PE-conjugated anti-TLR1 (GD2.F4), CD80 (2D10.4), and CD86 (IT2.2) from eBioscience, USA. Appropriate isotype controls for flow cytometry, mouse anti-CD11b (238439) and goat anti-IL-1β (AF-201-NA) was purchased from R&D Systems, USA. Mouse anti-CD47 (B6H12) and anti-CD81 (5A6), rabbit polyclonal anti-CD36 (H-300), anti-β-actin (N-21), goat anti-mouse IgG-HRP and rabbit anti-goat IgG-HRP antibodies were from Santa Cruz Biotechnology, USA. HRP-conjugated mouse anti-rabbit IgG (L) antibodies were from Jackson ImmunoResearch, USA. HRP-conjugated goat anti-rabbit IgG (H + L) antibodies, Precision Protein StrepTactin-HRP Conjugate and Precision Plus Protein Western C standards were purchased from Bio-Rad, USA. Human IL-10 ELISA Set, human TNF ELISA Set and human IL-1β ELISA Set II were from BD Biosciences, USA. Pierce™ LDH Cytotoxicity Assay Kit was purchased from Thermo Scientific, USA.

### Statistical Analysis

All experiments were performed with macrophages derived from at least three different donors each in duplicated or triplicated cultures (*n* = number of cultures). GraphPad Prism (GraphPad Software, USA) and Origin 8.1 (OriginLab, USA) software were used for statistical analysis. Data are given as mean ± SD, unless indicated otherwise. Statistical analysis was performed using Student's *t*-test to evaluate differences between two groups, and ANOVA for multiple comparisons. A statistically significant difference was assumed at *p* < 0.05.

## Results

### Phenotype Characteristics of hMDFCs Cultured With oxLDLs for Prolonged Time

**Figure 2:**
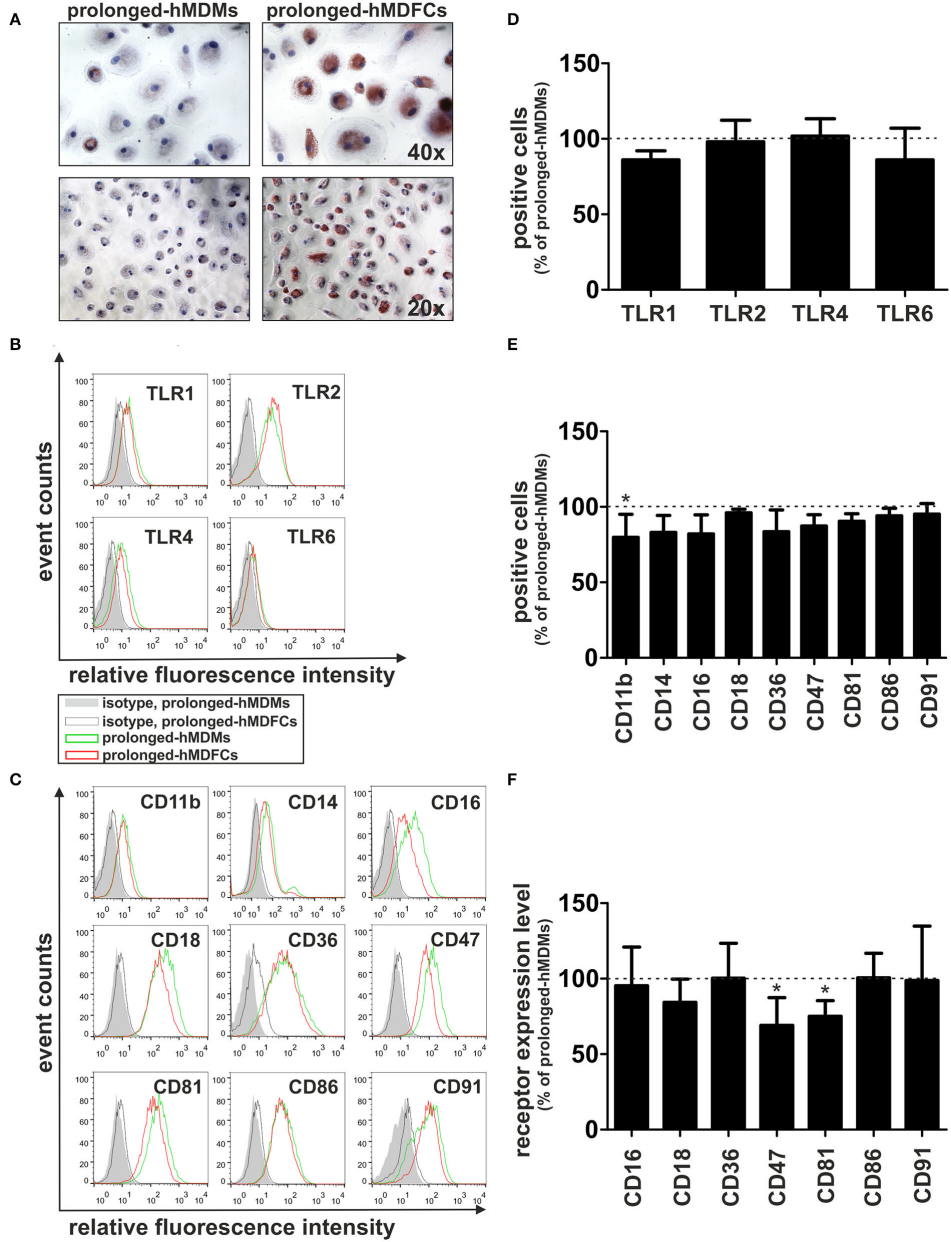
Effect of prolonged culture with oxLDLs on the phenotype of hMDMs. Adherent monocytes were cultured in HS medium for 5 days to allow macrophage differentiation. Since then, the cells were maintained in HS medium (prolonged-hMDMs) or HS medium supplemented with 5 μg/mL oxLDLs (prolonged-hMDFCs) for the total time period of 30 days. **(A)** The extent of lipid accumulation was assessed by Oil Red O staining. The cells were counterstained with hematoxylin. Representative staining images are shown. **(B,C)** Flow cytometric analysis of surface receptors expression in prolonged-hMDMs and hMDFCs. The cells were stained with monoclonal antibodies or appropriate isotype controls. Data are displayed in the form of histogram overlays using % Max option, which scales each population curve to mode 100%. A representative example out of three donors is shown. Positive population was identified using Overtone subtraction technique **(D,E)** and relative level of receptor expression was determined by MFI values of positive population **(F)**. Results for prolonged-hMDFCs are expressed as a ratio to corresponding prolonged-hMDMs (100% indicated with dashed horizontal line). Raw data of receptors expression (% of positive cells and MFI) on prolonged-hMDMs and hMDFCs are presented in Supplementary Tables 1, 2. Values are the means ± SD from at least three independent experiments (each in triplicate, *n* ≥ 9). *p < 0.05 vs. hMDMs.

To obtain prolonged-hMDFCs, human peripheral blood monocytes were cultured for 30 days in the constant presence of 5 μg/mL oxLDLs since the 5th day of differentiation. Monocytes isolated from the same donor and cultured in the absence of oxLDLs served as control prolonged-hMDMs. The Oil red O staining demonstrated that the prolonged-hMDFCs accumulated cholesterol in the form of large lipid droplets in the cytoplasm or dispersed deposits surrounding the nucleus (Figure 2A).

Flow cytometry analysis revealed high autofluorescence of prolonged-hMDFCs which was quenched with crystal violet according to the method described for alveolar macrophages and immature dendritic cells (27, 28). Comparative flow cytometry analysis of cell-surface expression of TLRs demonstrated no significant differences between hMDMs and hMDFCs cultured for prolonged time (Figures 2B,D and Supplementary Table 1). hMDFCs expressed significantly less CD47, CD81, and CD11b, while only a slight decrease was observed for CD14, CD16, and CD36 (Figures 2C,E,F, Supplementary Tables 1, 2). Both cell populations expressed comparable levels of CD18, CD86, and CD91 (Figures 2C,E). Neither hMDMs nor hMDFCs expressed DC-SIGN or CD80 markers (data not shown).

### Total Cellular Expression of Receptors in Prolonged-hMDFCs

**Figure 3:**
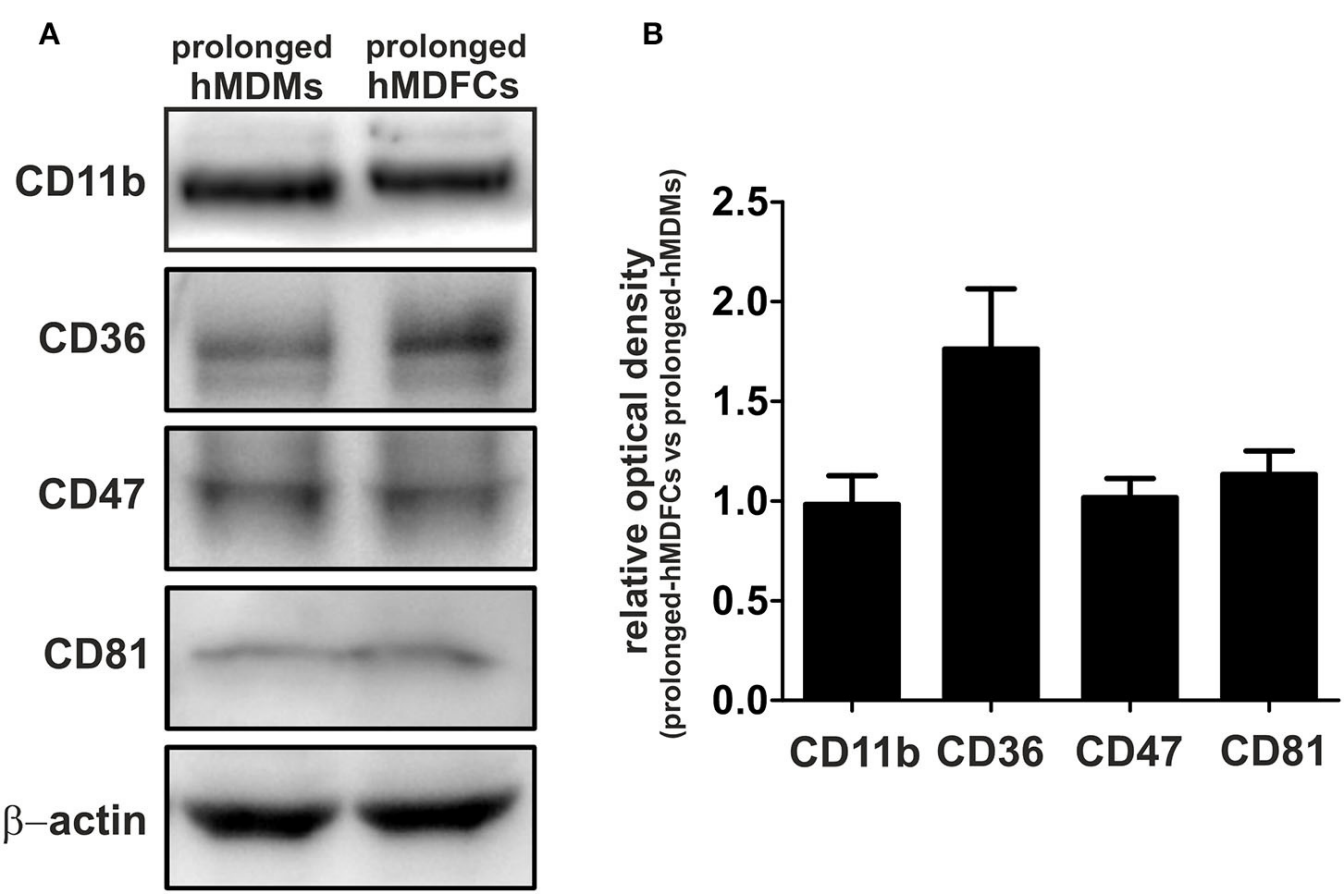
Western blot analysis of CD11b, CD36, CD47, and CD81 in prolonged- hMDMs and hMDFCs. Whole-cell lysates (25 μg) from prolonged- hMDMs and hMDFCs were analyzed using immunoblotting. Enhanced chemiluminescence signal was detected with Bio-Rad ChemiDoc XRS+ system. β-actin level was determined as a loading control. **(A)** Images shown are representative of four independent experiments (donors). **(B)** Quantification of protein levels. The optical density was measured for the bands of interest and normalized to β-actin signal. To compare expression levels of receptors, the relative optical density was calculated (prolonged-hMDFCs vs. corresponding prolonged-hMDMs). Results represents the mean ± SD from four independent experiments. Due to semi-quantitative nature of measurements, statistical analysis was not performed.

To verify flow cytometry results, we evaluated total cellular expression of proteins which surface expression was mostly modified in hMDFCs, i.e., CD47, CD81, and CD11b. We also analyzed the expression of CD36—a scavenger receptor that mediates uptake of oxLDLs as well as assists in diacylated lipopeptides recognition by TLR2/TLR6 heterodimer. Lysates from non-stimulated prolonged-hMDMs and prolonged-hMDFCs were subjected to Western blot analysis (Figure 3). We did not observe considerable differences in total expression of CD11b, CD47, and CD81 although their surface expression was significantly reduced in hMDFCs. Moreover, total amount of CD36 was slightly increased in hMDFCs while its surface expression remained unchanged.

### Spontaneous Secretion of Proteins by Prolonged-hMDFCs

**Figure 4:**
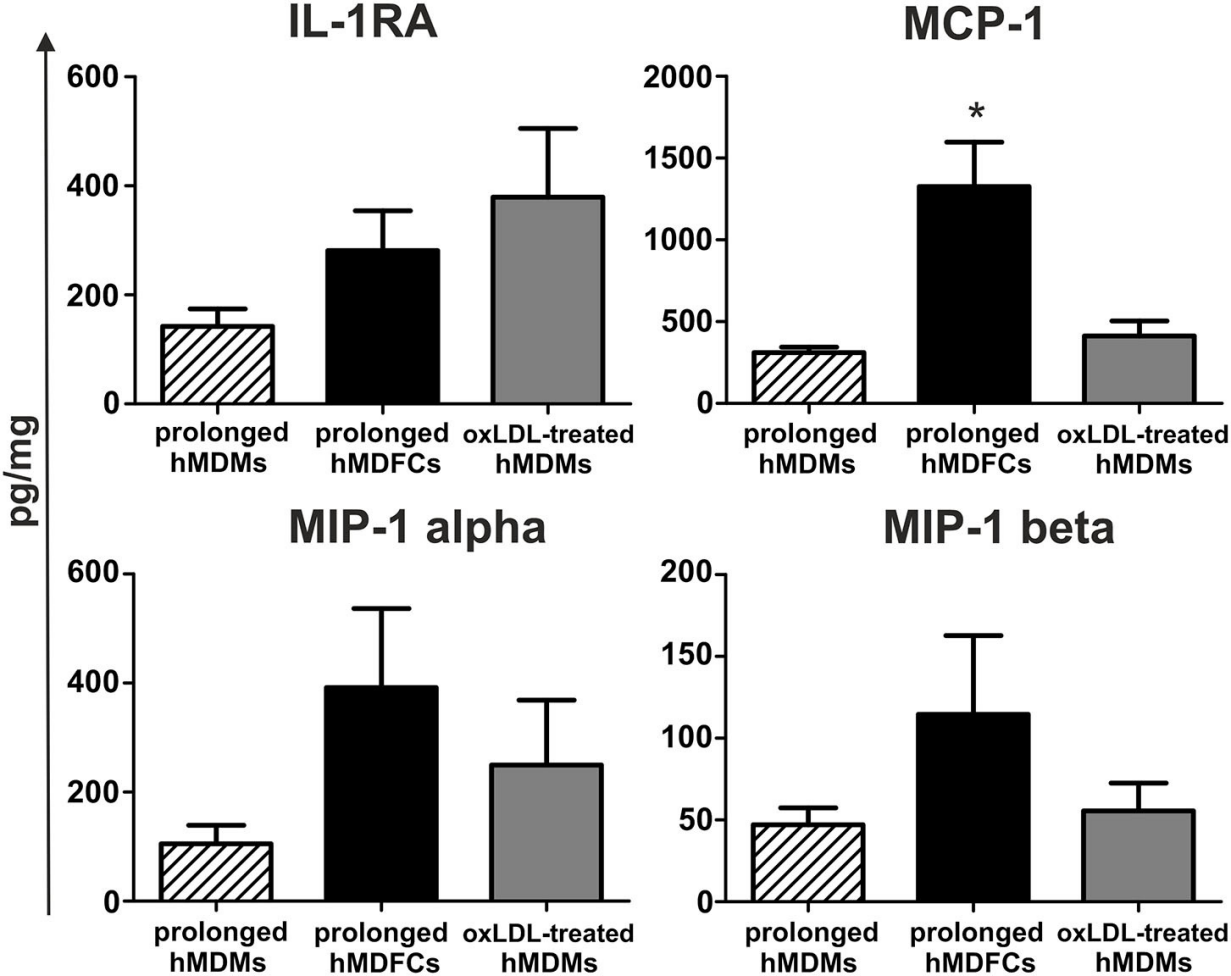
Spontaneous secretion of proteins by prolonged-hMDFCs. Prolonged- hMDMs and hMDFCs were obtained as indicated in Materials and Methods, and Figure 1. The cells were then incubated for 24 h in RPMI 1640 with 2% HS (prolonged-hMDMs) or RPMI 1640 with 2% HS, additionally supplemented with 5 μg/mL oxLDLs (prolonged-hMDFCs, oxLDL-treated-hMDMs). Levels of cytokines in supernatants were determined by a multiplex immunoassay. Values were normalized to protein concentration in corresponding cell cultures. Data represents production of cytokines secreted consistently at the measurable level. Bars and error bars depict means ± SD of at least three independent experiments (each in triplicate, *n* ≥ 9). *p < 0.05.

Next, we established a basal cytokine secretion by non-stimulated hMDFCs and hMDMs cultured for prolonged time. Because the spontaneous cytokine secretion by hMDFCs was determined for cells cultured constantly with oxLDLs, we prepared additional control that is prolonged-hMDMs exposed to oxLDLs only for 24 h before the measurement (oxLDL-treated-hMDMs). Cytokines were determined in supernatants using Luminex® assay. Neither hMDMs nor hMDFCs produced measurable levels of pro-inflammatory interleukins (Supplementary Table 3) and a few chemokines and IL-1RA were detected (Figure 4). In comparison with prolonged-hMDMs and oxLDL-treated-hMDMs, we noted a significant increase in MCP-1, MIP-1 alpha, and MIP-1 beta secretion by hMDFCs. It suggests that lipid-loading may favor the recruitment of other leukocytes to the niches occupied by foam cells.

### TLR-Induced Cytokine Production by Prolonged-hMDFCs

The primary aim of this study was to compare the ability of hMDMs and hMDFCs cultured for prolonged time to respond to PAMPs. For this purpose, after 30 days of culture, both cell types were stimulated with defined PAMPs molecules specific for TLR2 heterodimers (TLR1/2 or TLR2/6), TLR3, TLR4, TLR7, and TLR8. Alternatively, macrophages were treated with heat inactivated *P. gingivalis*. Supernatants were collected after 24 h and cytokines were analyzed with Luminex®. The mean concentrations of proteins are presented in Supplementary Table 3. Additionally we verified some outcomes of Luminex® assays through ELISA measurements of selected interleukins secreted by macrophages—IL-10, TNF, and IL-6—receiving consistent results.

**Figure 5:**
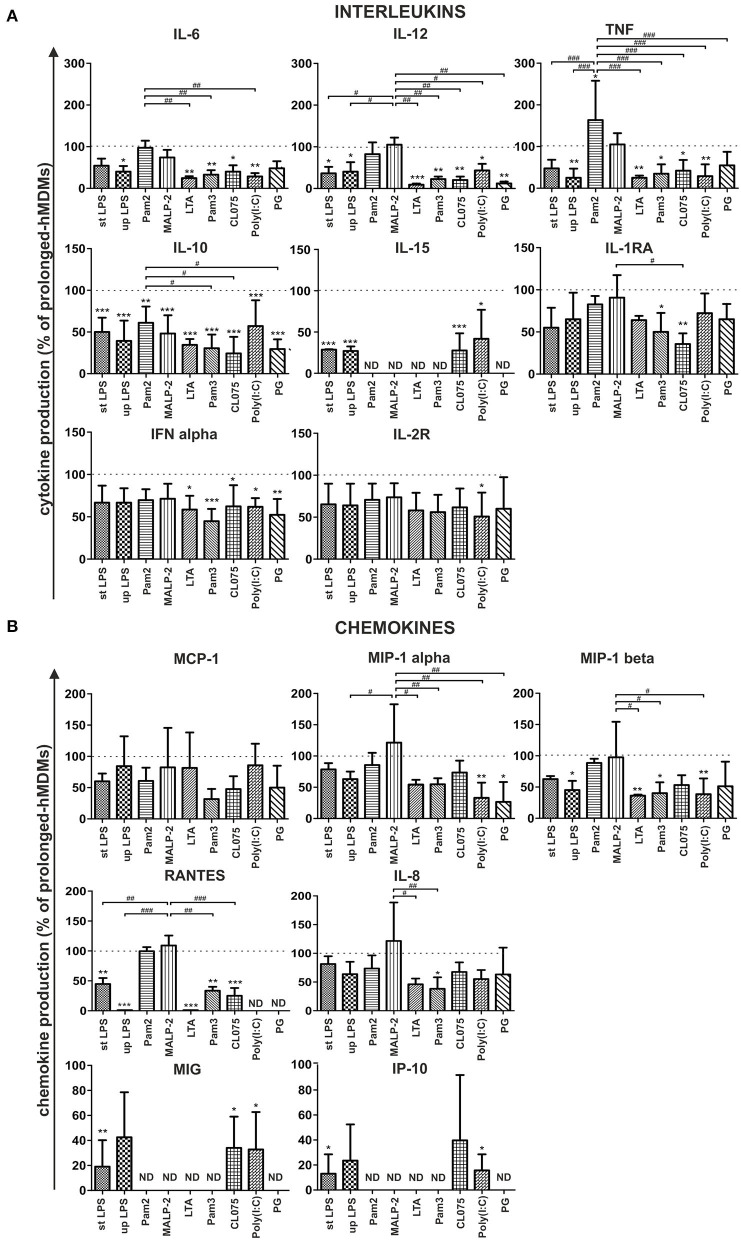
Effect of prolonged culture with oxLDLs on PAMPs-induced cytokine **(A)** and chemokine **(B)** production. Prolonged-hMDMs and hMDFCs were obtained as indicated in Materials and Methods, and Figure 1. Cells were then stimulated with different PAMPs for 24 h. Levels of cytokines in supernatants were determined by a multiplex immunoassay and normalized to protein concentration in corresponding cell cultures. Results for prolonged-hMDFCs were expressed as a percent of cytokine production by corresponding prolonged-hMDMs (100% indicated with dashed horizontal lines). Bars and error bars represent means ± SD from at least three independent experiments (each in triplicate, *n* ≥ 9). *p < 0.05, **p < 0.01, ***p < 0.001 vs. hMDMs; ^#^*p* < 0.05, ^##^*p* < 0.01, ^###^*p* < 0.01 vs. Pam2CSK4 or MALP-2 stimulation. Raw data of cytokine production are presented in Supplementary Table 3.

We noted that the profile of induced cytokines was similar in all experiments. Production of IL-15, MIG, IP-10 chemokines was induced only with TLR3, TLR4, and TLR7/8 ligands while RANTES were not detected upon LTA, Poly (I:C), and PG treatment. Cytokines below the lower limit of assay sensitivity (IL-1β, IL-2, IL-3, IL-4, IL-7, IL-13, IL-17, IFNγ, and EOTAXIN) were not included in further analysis. On account of considerable variability among donors, the protein secretion profiles of stimulated prolonged-hMDFCs are presented relative to corresponding hMDMs (Figure 5).

We observed that long-term (30 days) culture with oxLDLs resulted in moderate to significant alteration of both—interleukin and chemokine family secretion, in a PAMP- and TLR-dependent manner (Figure 5). Specifically, following activation of TLR4 (with ultrapure and standard *E. coli* LPS) prolonged-hMDFCs produced almost all of the measured molecules at lower level, with the exception of MCP-1, MIP-1 alpha, and IL-8. We also noted a significant inhibition of interleukin and, to a lesser extent, chemokine release after triggering receptors residing in endosomal compartments—TLR7/8 with a base analog CL075 and TLR3 with Poly (I:C). hMDFCs exposed to a complex structure (heat—killed bacteria *P. gingivalis*) secreted considerably lower amounts of IL-10, IL-12, IFN alpha, and MIP-1 alpha than hMDMs. Surprisingly, the response of prolonged-hMDFCs to TLR2/6 heterodimer agonists (diacylated lipopeptides—Pam2CSK4 and particularly MALP-2) remained mostly unaffected or even enhanced in case of proinflammatory cytokines and the majority of analyzed chemokines. However, the secretome response of these cells to other TLR2 ligands—LTA and Pam3CSK4 was severely inhibited with exception of MCP-1 for LTA.

Interestingly, lipid-loading exerted the most profound effect on anti-inflammatory and anti-atherosclerotic IL-10 secretion, which was significantly inhibited irrespective of ligand type used for cell stimulation. On the contrary, production of another anti-inflammatory mediator—IL-1RA was affected only after TLR1/2 and TLR7/8 triggering. Furthermore, MCP-1 and IL-8 release was unchanged or only slightly diminished in prolonged-hMDFCs stimulated with majority of PAMPs. Collectively, macrophages differentiated for a long time in the presence of oxLDLs alter their responsiveness to PAMPs in a manner specific for the challenging molecule and a member of TLR family involved.

**Figure 6:**
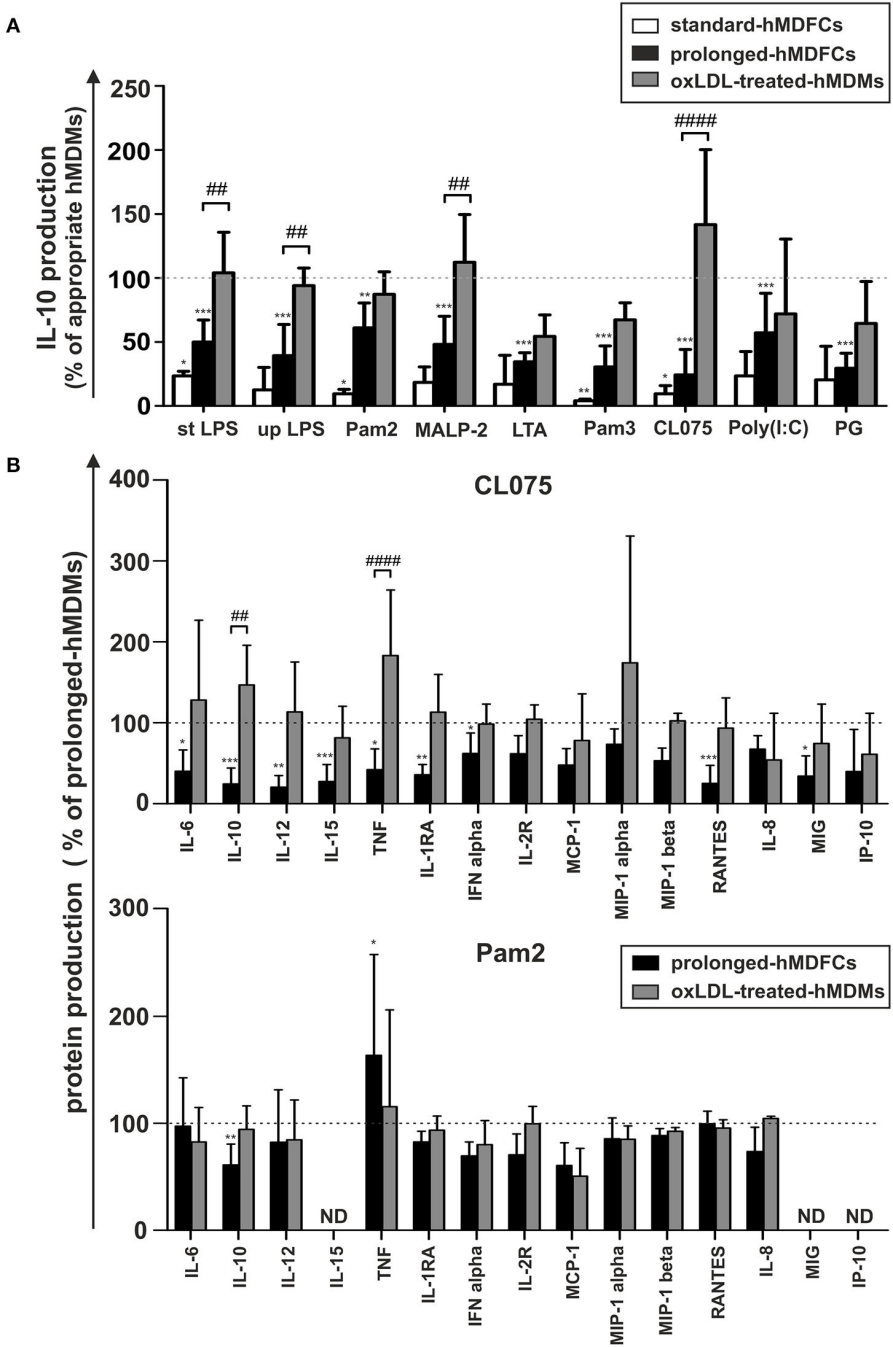
Comparison of IL-10 production by standard-hMDFCs, prolonged-hMDFCs, and oxLDL-treated-hMDMs **(A)**, and comparison of different cytokines production by prolonged-hMDFCs, and oxLDL-treated-hMDMs **(B)**. Standard/prolonged-hMDMs/hMDFCs were obtained as indicated in Materials and Methods, and Figure 1. Cells were then stimulated with different PAMPs for 24 h. Levels of cytokines in supernatants were determined by a multiplex immunoassay and/or ELISA (IL-10 and TNF) and normalized to protein content in corresponding cell cultures. Results for hMDFCs and oxLDL-treated-hMDMs were expressed as a percent of cytokine production by corresponding standard- or prolonged-hMDMs (100% indicated with dashed horizontal lines). Bars and error bars represent means ± SD from at least three independent experiments (each in triplicate, *n* ≥ 9). *p < 0.05, **p < 0.01, ***p < 0.001 vs. hMDMs, ^##^*p* < 0.01, ^####^*p* < 0.001 hMDFCs vs. oxLDL-treated hMDMs.

In this study, we also compared hMDFCs differentiated in our model, i.e., 30 days in the presence of low concentration of oxLDLs (prolonged-hMDFCs) with the foam macrophages obtained by the most common protocol, i.e., 3-days differentiation in the presence of oxLDLs supplied in 10-fold higher concentrations (standard-hMDFCs). Irrespective of cell type the extent of lipid loading was similar (Supplementary Figure 1). Also, IL-10 production was decreased in both—standard- and prolonged-hMDFCs as compared to corresponding hMDMs (Figure 6A). Nevertheless, the increased secretion of TNF induced by Pam2CSK4 and MALP-2 was observed only as a result of prolonged exposition to oxLDLs (data not shown). The decrease of IL-10 release by stimulated hMDFCs regardless of PAMPs can result from lipid accumulation in the cytoplasm or from oxLDL interference at the time of TLR triggering. Accordingly, we compared TLR-induced IL-10 production of hMDFCs with that of hMDMs treated with oxLDLs exclusively during cell stimulation—oxLDL-treated-hMDMs (Figure 6A). As described in Materials and Methods cells were stimulated in medium containing oxLDLs, the supernatants were collected after 24 h and cytokines were measured by Luminex® and/or ELISA. The results were expressed as a percent of cytokine production by corresponding hMDMs. As shown in Figure 6A, the presence of oxLDLs during PAMPs stimulation of TLR4, TLR7/8, and TLR2/6 (Pam2CSK4, MALP-2) did not inhibit IL-10 secretion. Notably, upon CL075 induction, the IL-10 production by oxLDL-treated cells was even higher than by prolonged-hMDMs. In case of LTA, Pam3CSK4, and PG treatment we observed decrease of IL-10 release in both cell cultures indicating that final IL-10 production is influenced by cytoplasmic accumulation of oxLDLs as well as their presence in medium. Still, the IL-10 suppression was more pronounced in hMDFCs.

To further correlate the profile of cytokine response to distinct PAMPs with the cellular oxLDL accumulation, we analogously compared secretion of other proteins in prolonged-hMDFCs and oxLDL-treated-hMDMs (Figure 6B). Triggering of endosomal TLR7/8 with CL075 was strongly dependent on lipid-loading. In sharp contrast with the hMDFCs from prolonged culture, the presence of oxLDLs only at the moment of stimulation did not suppress the macrophage secretome. Moreover, in oxLDL-treated cells the production of IL-10 and TNF was even enhanced. Following stimulation with Pam2CSK4 only the long-term lipid-loading led to significant IL-10 inhibition and TNF upregulation. For other PAMPs we noted rather comparable pattern of secretome modifications in prolonged-hMDFCs showing a slight tendency to stronger reduction of protein production in comparison with oxLDL- treated counterparts (data not shown).

The data indicate that, in contrast to other cytokines, IL-10 secretion in response to pathogenic stimuli is affected predominantly by lipid loading of macrophages. It also suggests that the plasma membrane TLR signaling is modulated by both lipid accumulation and oxLDLs presence at the moment of PAMPs recognition while endosomal TLR signaling is modulated mainly by the intracellular lipids.

### Pyroptosis and IL-1β Secretion by Standard- and Prolonged hMDFCs

**Figure 7:**
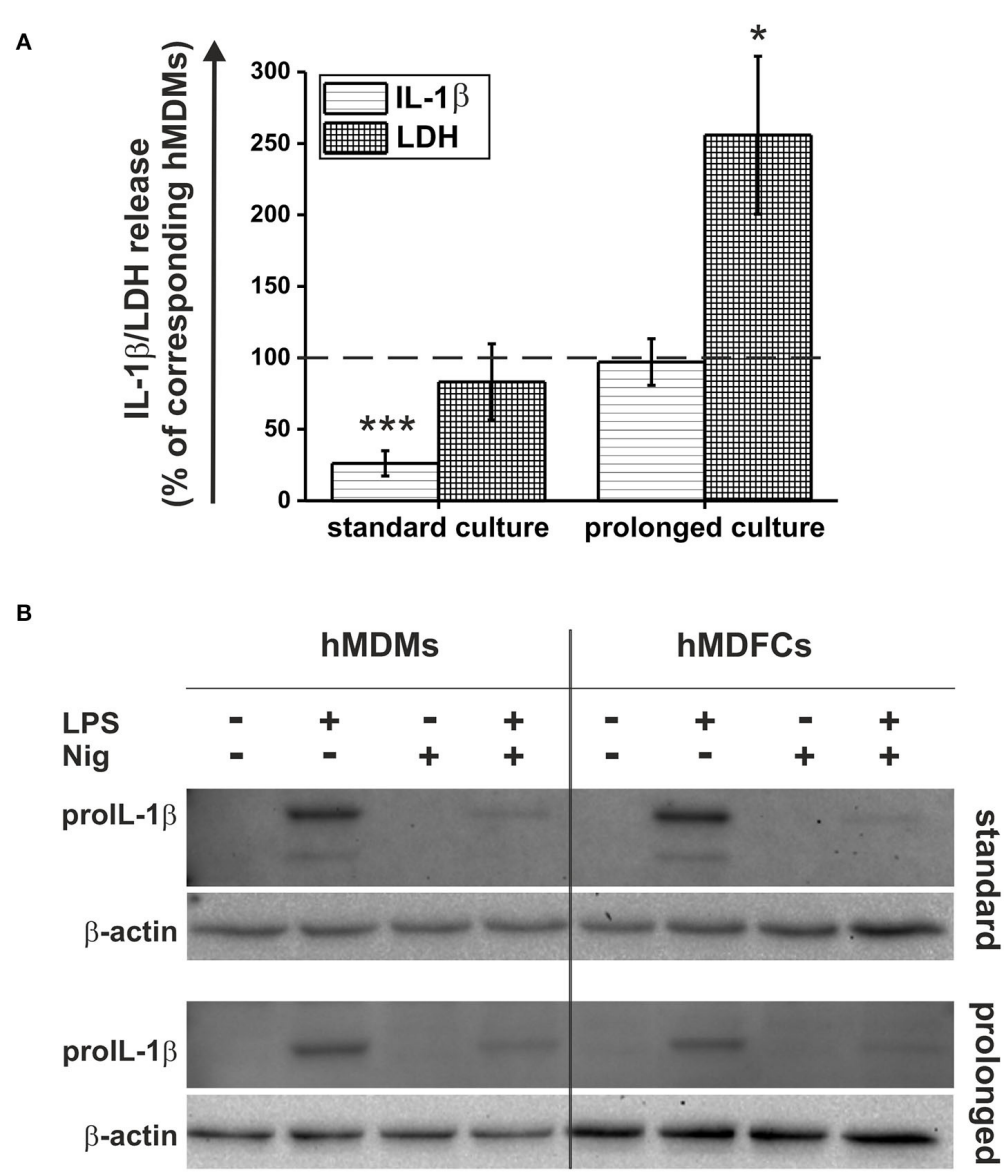
Pyroptosis and IL-1β secretion by hMDFCs obtained by standard and prolonged cell culture. Standard/prolonged-hMDMs/hMDFCs were obtained as indicated in Materials and Methods, and Figure 1. Cells were stimulated with 1 μg/mL stLPS for 4 h and 10 μM nigericin for next 20 h. **(A)** Levels of IL-1β and LDH activity in supernatants were determined by ELISA and Pierce™ LDH Cytotoxicity Assay Kit, respectively. Results for hMDFCs were expressed as a percent of IL-1β secretion and LDH release by corresponding standard- and prolonged hMDMs (100% indicated with dashed horizontal lines). Bars and error bars represent means ± SEM from seven independent experiments (each in duplicate, *n* = 14). *p < 0.05, ***p < 0.001 vs. hMDMs. Raw data of IL-1β production and LDH release were shown in Supplementary Figure 2. **(B)** ProIL-1β expression was determined in whole-cell lysates from hMDMs and hMDFCs using immunoblotting. Enhanced chemiluminescence signal was detected with Bio-Rad ChemiDoc XRS+ system. β-actin level was determined as a loading control. Images shown are representative of three independent experiments (donors).

In this study, we compared the activation of NLRP3 inflammasome in hMDMs and hMDFCs differentiated in standard and prolonged culture. For this purpose, the cells differentiated as outlined in Figure 1 were primed with *E. coli* LPS for 4 h and then stimulated with nigericin for additional 20 h. The activation of NLRP3 inflammasome was evaluated by two parameters: IL-1β levels and LDH activity assayed in extracellular medium. Raw data obtained from seven independent experiments were shown in Supplementary Figure 2. It was found that for each cell type, LPS and nigericin stimulation led to both: IL-1β secretion and cell death occurring with the loss of plasma membrane integrity and release of LDH. However, we noticed differences in the response of foam cells obtained in accordance with the two analyzed models. In Figure 7A LPS and nigericin-induced release of IL-1β and LDH from hMDFCs were presented relative to corresponding hMDMs. Standard-hMDFCs were characterized by significantly decreased production of IL-1β in comparison with hMDMs counterparts, which cannot be ascribed to lower cell viability. The opposite situation was revealed for a prolonged culture—foam cells were able to produce IL-1β at a similar level as prolonged-hMDMs, however, a greater cytotoxic effect was observed.

Considering the fact that standard-hMDFCs were characterized by clearly lower IL-1β secretion (Figure 7A), we evaluated total cellular expression of proIL-1β in lysates from hMDMs and hMDFCs by Western Blot analysis (Figure 7B). In non-stimulated cells and those treated only with nigericin proIL-1β was not detected. This protein appeared in a significant amount after priming with LPS, and declined after stimulation with nigericin. That is, most likely, a result of the NLRP3 inflammasome activation that facilitates caspase-1 autoactivation and subsequent proteolytic cleavage of proIL-1β into a mature form that is released from the cell. Importantly, we did not observe considerable differences in LPS-induced proIL-1β expression between hMDMs and hMDFCs in both standard and prolonged cultures. Additionally, despite the low band intensity in cells stimulated with LPS and nigericin, it was also visible that in hMDMs and hMDFCs the amount of proIL-1β present in the cells after activation of the NLRP3 inflammasome was comparable.

**Figure 8:**
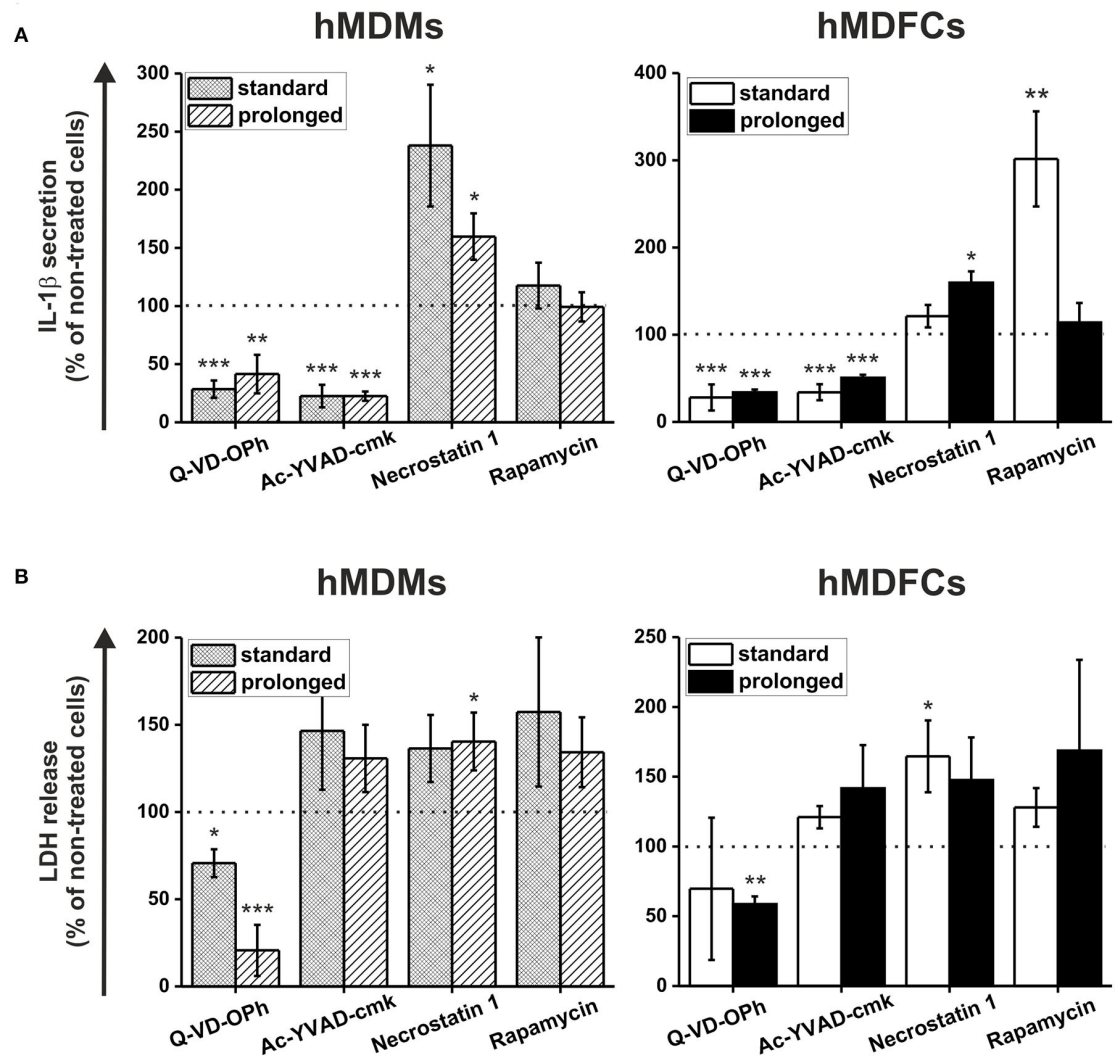
The effect of caspases, RIP1 kinase and mTOR inhibition on pyroptosis and IL-1β secretion by hMDFCs obtained by standard and prolonged cell culture. Standard/prolonged-hMDMs/hMDFCs were obtained as indicated in Materials and Methods, and Figure 1. Cells were primed with stLPS (1μg/mL) for 4 h and then stimulated with nigericin (10 μM) for 20 h with or without caspase inhibitors (Q-VD-OPh or Ac-YVAD-cmk), RIP1 kinase inhibitor (necrostatin-1) or mTOR inhibitor (rapamycin) pretreatment as described in Materials and Methods. Levels of IL-1β **(A)** and LDH **(B)** in supernatants were determined by ELISA and Pierce™ LDH Cytotoxicity Assay Kit, respectively. Results were expressed as a percent of IL-1β secretion and LDH release by cells stimulated without inhibitors—non-treated cells (100% indicated with dashed horizontal lines). Bars and error bars represent means ± SEM from four (Q-VD-OPh) or five (others) independent experiments (each in duplicate, *n* ≥ 8). *p < 0.05, **p < 0.01, ***p < 0.001 vs. non-treated cells. Raw data of IL-1β production and LDH release were shown in Supplementary Figure 3.

In the next set of experiments standard- and prolonged- hMDMs and hMDFCs were pretreated before activation of NLRP3 inflammasome with pan-caspase inhibitor (Q-VD-OPh), selective caspase-1 inhibitor (Ac-YVAD-cmk), RIP1 kinase inhibitor (necrostatin-1) or mTOR inhibitor (rapamycin). Raw data of IL-1β secretion and LDH release was shown in Supplementary Figure 3. Figure 8 depicts the obtained results expressed as a percent of IL-1β and LDH release by cells non-treated with inhibitors. As expected, caspase-1 inhibitor clearly reduced IL-1β secretion in hMDMs and hMDFCs to 15–22 and 34–50%, respectively. Surprisingly, in all four types of cells inhibition of caspase-1 activity had no noticeable effect on the cell death. IL-1β secretion was also significantly reduced by pan-caspase inhibitor. Interestingly, in contrast to selective caspase-1 inhibition, pretreatment with Q-VD-OPh influenced the cell death decreasing release of LDH from prolonged- hMDMs and hMDFCs. To test if necroptosis is involved in LDH release, we inhibited RIP1 kinase activity by the chemical inhibitor necrostatin-1 and we observed slightly increased plasma membrane permeabilization for LDH. Moreover, IL-1β secretion was strongly augmented in standard-hMDMs and to a lesser degree in both prolonged- hMDMs and hMDFCs following pretreatment with necrostatin-1. A growing body of evidence indicates that autophagic flux becomes defective in macrophages of advanced atherosclerotic plaques, and that treatment with an autophagy inducer can be exploited as a potential strategy to prevent plaque formation and destabilization (8). In endothelial cells cultured *in vitro* oxLDL-induced pyroptosis is restricted by autophagy (29). Consequently, we speculated whether, in our foam macrophages, pyroptosis can be counterbalanced by induction of autophagy. Autophagy induction, as a result of mTOR inhibition by rapamycin, had minor effect on IL-1β secretion with the exception of standard-hMDFCs where IL-1β production was increased almost 3-fold. Cells death was generally augmented in both macrophages and foam cells, most apparently in prolonged-hMDFCs.

## Discussion

The relationship between continuous accumulation of foam cells in atherosclerotic plaques and maintenance of chronic inflammation during atherogenesis is not fully understood. *In vitro* approaches to study the inflammatory status of oxidized lipid-loaded macrophages yielded controversial results. Whereas, some studies have reported minimal or no effects (30, 31), others showed pro- (32, 33) or anti-inflammatory (34–41) effects of loading with oxLDLs. This may be due to differences in the experimental conditions, including oxLDL concentration, oxidation levels of LDL particles and the duration of oxLDL treatment. In this study, human monocyte-derived macrophages were constantly exposed to purified oxLDLs for 30 days. Such obtained prolonged-hMDFCs displayed the characteristic macrophage-like morphology, with visible accumulation of cytoplasmic lipid droplets and typical macrophage phenotype (Figure 2, Supplementary Figure 1). We did not observe any cytotoxic effects of prolonged oxLDL loading of macrophages (not shown). In response to PAMPs both prolonged- hMDMs and hMDFCs produced identical set of interleukins and chemokines (Supplementary Table 3). While the overall pattern of secreted proteins remained unchanged, the cytokine release by prolonged-hMDFCs in response to PAMPs was mainly decreased (Figure 4). Notably, the long-term oxLDL loading was not a proinflammatory event but rather it reduced the inflammatory response when hMDFCs were stimulated with LPS, Poly (I:C), and Pam3CSK4—ligands of TLR4, TLR3, and TLR2/1, respectively (Figure 4). Inhibition has been observed for the majority of cytokines and RANTES, MIP, IP-10 chemokines by prolonged-hMDFCs following stimulation of TLR2, TLR4 and endosomal TLRs. It has been shown that human macrophages stimulated with high doses of LPS (500 ng/mL) in the presence of oxLDLs did not change (42) or downregulate (34) the production of TNF and IL-6. In contrast, Bekkering et al. reported increased production of proinflammatory cytokines by human monocytes briefly pre-exposed to a low concentration of oxLDLs, suggesting epigenetic reprogramming of monocytes (43). In our settings, IL-6, IL-8, IL-12, and TNF were not induced upon LPS stimulation either in oxLDL-treated-hMDMs or in prolonged-hMDFCs (Figure 5, Supplementary Table 3, and data not shown). In contrast to the suppression of LPS signaling, the stimulation of proinflammatory proteins with Pam2CSK4 or MALP-2 (ligands of TLR2/6) was enhanced in prolonged-hMDFCs (Figure 5). That was not observed for LTA or Pam3CSK4 (ligands of TLR1/2), although it is known that both heterodimers, TLR1/2 and TLR2/6, activate the same signaling pathways (44). Thus, our results reveal a novel branching in downstream responses to heterodimer-specific TLR2 stimulation in foam cells. The distinct downstream effects following the stimulation of lipid-laden macrophages through TLR2/1, TLR2/4, or TLR2/6 may result from activation of divergent signaling pathways, but also from different composition of signaling complexes involved in their detection. So far, however, the same accessory components have been described for optimal recognition of either LTA or diacylated lipopeptides including LBP, CD14, and CD36 (45–47). Recognition of triacylated Pam3CSK4 has also been shown to engage CD14, LBP and less probably CD36 (46, 48, 49). Active component of oxLDLs, oxPAPC, has been shown to compete with TLR ligands for CD14 and LBP binding, which leads to diminished signaling pathways activation and cytokine response (31, 50–52). On the other hand, up to 90% of circulating oxLDLs can be found in the form of oxLDL immune complexes (53) and it was shown that these complexes bind FcγRI on a human macrophage cell line and induce the secretion of proinflammatory cytokines (54). In a more recent study their role in priming of NLRP3 inflammasome and IL-1β production was also shown (55). The ability of oxLDLs to enable cross-talk between FcγR and TLR is likely facilitated by the tight clustering of TLRs and FcγRs in glycoprotein microdomains (56). In our cell culture settings, a fraction of oxLDLs could also form immune complexes with Abs from supplemented human serum as it contains substantial titers of anti-oxLDL antibodies (57). We have been particularly interested whether prolonged-hMDFCs retain the ability to produce anti-inflammatory proteins. We observed that the production of IL-10 by these cells was considerably inhibited irrespectively of the inducing PAMP (Figure 5A). Apparently, the IL-10 production was negatively regulated by accumulated cytoplasmic lipids but not by oxLDLs present in medium during TLR interaction with PAMPs (Figure 6A). We also noted that the production of IL-1RA, an IL-10-dependent protein typical for M2 macrophages, was significantly lower only in case of TLR1/2 and TLR7/8 stimulation (Figure 5A). Additionally, non-stimulated oxLDL-treated-hMDMs and prolonged-hMDFCs spontaneously secreted more IL-1RA than corresponding prolonged-hMDMs (Figure 4). The response of endosomal receptors TLR7/8 was dramatically inhibited in prolonged-hMDFCs but not in oxLDL-treated-hMDMs (Figure 6B). It suggests that the endosomal TLR signaling is modulated mainly by the intracellular lipids. The response of prolonged-hMDFCs to the stimulation of membrane TLRs and another endosomal receptor—TLR3 was apparently regulated by both the cytoplasmic lipid deposits and extracellular oxLDLs, depending on structural characteristic of the ligand (Figure 6 and data not shown). We verified that the PAMPs-induced secretome of prolonged-hMDFCs is not caused by the change of surface expression of TLR1, TLR2, TLR4, and TLR6 (Figures 2B,D). Further cytometric analysis revealed decreased expression of surface CD81, CD47, and CD11b on hMDFCs (Figures 2C,E,F). Western blot analysis, however, did not reveal any differences in total cellular lysates of prolonged-hMDMs and hMDFCs (Figure 3). The diminished surface exposition of CD47 and CD81 may result from their sequestration inside lipid-laden cells or direct interactions with other surface proteins on the plasma membranes which make them less accessible to antibodies by steric hindrance. CD81 is as a member of tetraspanin family, known to facilitate formation of multiple molecular complexes in membrane microdomains. Heit et al. demonstrated that CD81 resides in heterogenous CD36 multimolecular complexes containing another tetraspanin CD9 and integrins β1/β2 (58).

CD47, also known as a integrin-associated protein, is a widely expressed cell surface signaling receptor that regulates cell viability and responses to stress. CD47 lacks a substantial cytoplasmic signaling domain, but several cytoplasmic binding partners have been identified. Additionally, lateral interactions of CD47 with other membrane receptors play important roles in mediating signaling, that is initiated by the binding of thrombospondin-1 (59).

To obtain prolonged-hMDFCs we used low concentration of oxLDLs (5 μg/mL) yet the concentrations of modified LDLs in the vessel wall may be considerably higher (60). On the other hand, there is no accepted gold standard for *in vitro* preparation of oxLDLs (61). In this work we used copper sulfate-derived oxLDLs which is the most widely accepted model of highly oxidized LDLs. Such lipoproteins are not fully physiological molecules and probably do not entirely represent oxidatively modified LDL *in vivo*. Nevertheless, copper-treated LDL resemble naturally occurring oxLDLs and antibodies raised against such obtained oxLDLs successfully detect epitopes of human oxLDLs (62). OxLDL preparation used in indicated concentration was not cytotoxic to the cells as determined by LDH assay and cytometric analyses (data not shown). Our oxLDL preparations did not stimulate IL-6, IL-12, or TNF production in monocytes or in macrophages (data not shown). Moreover, we did not detect IL-1β in supernatants of both non-stimulated and PAMPs-triggered foam-cells (Supplementary Figure 2). This is in line with Netea et al. who proved that human macrophages did not secrete IL-1β upon TLR stimulation alone (63). We noted, however, significant increase in MCP-1 and slight induction of MIP-1 alpha, MIP-1 beta secretion by non-stimulated prolonged-hMDFCs (Figure 4). These findings indicate that lipid accumulation may favor chemotactic recruitment of other cell types into atherosclerotic lesion. Short incubation of hMDMs with oxLDLs induced also a minor increase of IL-1RA secretion (Figure 4).

Results of our study indicate that although hMDFCs residing in oxLDL—rich environment retain the ability to recognize and respond to a variety of pathogenic structures, they are generally less capable of strong inflammatory reaction. Local disregulation of proinflammatory mediators, as reported here for the majority of PAMPs, may lead to generation of chronic, non-resolving inflammation characteristic for atherosclerosis. This process may be further perpetuated by lowered IL-10 production by these cells. The recognition of diacylated lipoproteins by prolonged-hMDFCs is potentially more proinflammatory than that of other PAMPs, however its biological significance is not yet clear. Although the mechanisms of oxLDL-mediated alteration of protein secretion by prolonged-hMDFCs have not been specified, this work provides an additional insight into the possible processes responsible for atherosclerotic plaque development.

This work has been based on two assumptions coming from the current state of cardiovascular science: (i) prolonged exposure of foam macrophages to oxLDLs will modify their response to PAMPs and (ii) induction of the major immunogenic cell death type—pyroptosis, will also be altered.

In recent years, more and more studies have been focusing on the links between atherosclerosis and inflammation (5, 16, 24, 64, 65), infectious diseases (66–68), and pyroptosis (8, 10, 69, 70)—a pro-inflammatory type of cell death dependent on the activation of inflammasome. Mounting evidence suggests that oxLDLs activate NLRP3 inflammasomes through lysosomal rupture and subsequent cathepsin release (10, 70), which, in turn, leads to cleavage and activation of procaspase-1 and pyroptosis. Our current investigation shows that oxLDLs act as a regulatory factor for IL-1β production and a caspase-dependent and -independent necrotic type cell death. Challenged by the standard LPS/nigericin, two-signal inflammasome stimulus, the prolonged-hMDFCs produced much lower absolute amounts of IL-1β than foam cells cultured in typical settings—standard-hMDFCs (Supplementary Figure 2), but higher when ratio of foam cell to macrophage counterpart was compared (Figure 7A). Some studies showed that oxLDLs induces inflammasome-mediated IL-1β production in BMDMs (35, 40, 41), however, some others have not confirmed this finding (55). This discrepancy is likely a time- and dose-dependent issue. Jiang et al. (40) observed IL-1β production by BMDMs treated with increasing concentrations of oxLDLs (25–200 μg/mL) for 12 h, performing most of the experiments with 200 μg/mL oxLDLs. Similarly, Liu et al. (41) used high concentrations of oxLDL (50–200 μg/mL) for 24 h. Although these high concentrations of oxLDLs evoke powerful responses from innate immune cells, they are at the upper extremum of physiological relevance. We decided to use 5 μg/mL oxLDLs for our studies to more closely mimic conditions *in vivo* (71, 72). In addition, the long (30 days) incubation period allowed time for the formation of cholesterol crystals, which is the primary mechanism by which oxLDLs activates the inflammasome. Studies by Sheedy et al. demonstrated that oxLDLs can be a second, activating signal for the inflammasome through a TLR4/TLR6/CD36 heterotrimer complex (35). This was largely facilitated by formation of cholesterol crystals and resulting lysosomal disruption. The investigators went on to suggest that oxLDLs could act as both—a priming and activating signal for the inflammasome via TLR ligation and cholesterol crystal formation, respectively. Although we cannot exclude such a possibility, we observed neither spontaneous production of IL-1β (Supplementary Figure 2) nor increased accumulation of proIL-1β (Figure 7B) in non-stimulated prolonged-hMDFCs.

Involvement of caspase-1 is supported by the observation that IL-1β production was decreased by caspase-1-specific inhibitor (Ac-YVAD-cmk), but not by necrostatin-1, a RIP-1 inhibitor (Figure 8A and Supplementary Figure 3). Interestingly, necrostatin-1 increased IL-1β production in hMDMs (and less in hMDFCs) as well as necrotic type cell death stimulated with LPS/nigericin (Figure 8 and Supplementary Figure 3). This may suggest that RIP1 activity is necessary to tame the excessive inflammasome activation in differentiating macrophages. Karunakaran et al. demonstrated that oxLDLs directly induce necroptosis in the absence of synthetic caspase inhibitors and that the expression of RIP3 in carotid plaques is higher than in disease-free control arteries (73). They also observed induction of RIP3 activity in murine BMDMs treated with oxLDLs *in vitro*. The results may explain weaker cytotoxicity of necrostatin-1 observed in our prolonged-hMDFCs (Figure 8). The induction of necroptosis by oxLDLs was found independent of inflammasome activation, because cells deficient in caspase-1 or treated with caspase-1 inhibitors underwent necroptotic cell death in response to oxLDLs to the same degree as wild-type or untreated cells (73). A particularly interesting feature reflected in prolonged-hMDFCs was enhanced sensitivity to rapamycin, an autophagy-inducing mTOR inhibitor. The rapamycin-treated hMDFCs displayed a necrotic type death which, as measured by LDH release, was disproportionately larger than IL-1β production (Figure 8 and Supplementary Figure 3). This is in contrast to hMDMs or standard-hMDFCs where the proportion of necrotic cells was moderate (Figure 8). Apparently, the prolonged-hMDFCs enter necrotic pathways other than pyroptosis more promptly than standard-hMDFCs. Furthermore, foam cells derived from THP-1 macrophages in 72 h culture did not respond to everolimus (an FDA-approved rapalog) with necrosis (74). Indeed, limited clinical data show substantial depletion of plaque macrophages by everolimus treatment (75), which may result from excessive necrosis without a corresponding production of IL-1β. Cumulatively, the data from short-term-induced foam cells demonstrated an important limitation of the model which does not fully reflect the cross-talk between different processes connected with cell death, particularly pyroptosis, necroptosis and autophagy. The coexistence of different cell death pathways and their mutual regulation in foam cells has not been extensively studied so far, but our present data suggest a gradual, multi-stage process of foam cell formation which is supported by incidental observations in primary human foam cells. In the initial stage of atherosclerosis the activation of mTOR signaling contributes to the formation of foam cells via enhancing the process from monocyte to macrophage. In later stages the activation of mTOR signaling promotes formation of fatty streaks and facilitates the formation of vulnerable plaques (76). In advanced lesions overexpression of MLKL (a necroptosis effector kinase) activates inflammation and inhibits autophagy flux (77).

In conclusion, the current study demonstrates that long-term exposure of macrophages to moderate amounts of oxLDLs lead to profound changes in their ability to respond to external stimuli without dramatic changes of phenotype. Particularly, we observed strong inhibition of cytokine and chemokine secretion upon TLR1/2, TLR4, and endosomal TLR stimulation. Surprisingly, the prolonged-hMDFCs response to diacylated lipopetides, which are ligands of TLR2/6, was not significantly changed. We also found that impairment of IL-10 release is strongly dependent on the extent of lipid loading by macrophages. Prolonged-hMDFCs responded to inflammasome activation with pyroptosis combined with other types of necrotic death. Such immunogenic response to inflammasome triggers was aggravated by specific inhibitors of apoptosis and necroptosis, and by stimulation of autophagy. These findings identify an important contribution of mature foam cells to innate immune responses that goes beyond their previous recognition and highlights necrotic cell death pathways as potential markers for atherosclerosis disease severity. Better understanding of the cross-talk between cell death pathways in atherosclerosis is an area of continued interest and warrants further study.

## Data Availability Statement

The datasets generated for this study are available on request to the corresponding author.

## Author Contributions

AD, AN, and MB performed the experiments. AN and MB participated in the manuscript preparation. CV provided the intellectual contribution and revised the manuscript. KG designed the study, wrote the manuscript, and coordinated the project. All authors contributed to the article and approved the submitted version.

## Conflict of Interest

The authors declare that the research was conducted in the absence of any commercial or financial relationships that could be construed as a potential conflict of interest.

## Abbreviations

DAMPs: damage-associated molecular patterns
hMDFCs: human monocyte-derived foam cells
hMDMs: human monocyte-derived macrophages
HS: human serum
IFN-α: interferon alpha
IL: interleukin
IL-1RA: interleukin-1 receptor antagonist
IL-2R: interleukin-2 receptor
IP-10: IFN-γ-Inducible Protein 10, CXCL10
LDL: low-density lipoproteins
LOX-1: lectin-like oxidized low-density lipoprotein (LDL) receptor-1
LTA: lipoteichoic acid
MALP-2: macrophage-activating lipopeptide 2
MARCO: macrophage receptor with collagenous structure
MCP-1: monocyte chemotactic protein-1
MFI: mean fluorescence intensity
MIP-1: macrophage inflammatory protein-1
oxLDLs: oxidized low-density lipoproteins
Pam2: synthetic lipopeptide, Pam2CysSerLys4
Pam3: synthetic lipopeptide, Pam3CysSerLys4
PAMPs: pathogen-associated molecular patterns
PBMCs: peripheral blood mononuclear cells
PG: *Porphyromonas gingivalis*
Poly (I:C): polyinosinic-polycytidylic acid
PRR: pattern recognition receptor
RANTES: Regulated on Activation Normal T*-*cell Expressed and Secreted
stLPS: standard lipopolysaccharide
TLR: Toll-like receptor
TNF: tumor necrosis factor
upLPS: ultra pure lipopolysaccharide.

## References

[1] World Health Organisation Cardiovascular Diseases. Geneva: WHO (2017).

[2] Berenson GS Srinivasan SR Bao W Newman WP Tracy RE Wattigney WA. Association between multiple cardiovascular risk factors and atherosclerosis in children and young adults. N Engl J Med. (1998) 338:1650–6. 10.1056/NEJM199806043382302 9614255

[3] Herrington W Lacey B Sherliker P Armitage J Lewington S. Epidemiology of atherosclerosis and the potential to reduce the global burden of atherothrombotic disease. Circ Res. (2016) 118:535–46. 10.1161/CIRCRESAHA.115.307611 26892956

[4] Tabas I Lichtman AH. Monocyte-macrophages and T cells in atherosclerosis. Immunity. (2017) 47:621–34. 10.1016/j.immuni.2017.09.008 29045897 PMC5747297

[5] Bäck M Yurdagul A Tabas I Öörni K Kovanen PT. Inflammation and its resolution in atherosclerosis: mediators and therapeutic opportunities. Nat Rev Cardiol. (2019) 16:389–406. 10.1038/s41569-019-0169-2 30846875 PMC6727648

[6] Moore KJ Sheedy FJ Fisher EA. Macrophages in atherosclerosis: a dynamic balance. Nat Rev Immunol. (2013) 13:709–21. 10.1038/nri3520 23995626 PMC4357520

[7] Tabas I Bornfeldt KE. Macrophage phenotype and function in different stages of atherosclerosis. Circ Res. (2016) 118:653–67. 10.1161/CIRCRESAHA.115.306256 26892964 PMC4762068

[8] Martinet W Coornaert I Puylaert P De Meyer GRY. Macrophage death as a pharmacological target in atherosclerosis. Front Pharmacol. (2019) 10:1–18. 10.3389/fphar.2019.00306 31019462 PMC6458279

[9] Tait SWG Ichim G Green DR. Die another way–non-apoptotic mechanisms of cell death. J Cell Sci. (2014) 127:2135–44. 10.1242/jcs.093575 24833670 PMC4021468

[10] Grebe A Hoss F Latz E. NLRP3 inflammasome and the IL-1 pathway in atherosclerosis. Circ Res. (2018) 122:1722–40. 10.1161/CIRCRESAHA.118.311362 29880500

[11] Winkels H Ehinger E Vassallo M Buscher K Dinh HQ Kobiyama K. Atlas of the immune cell repertoire in mouse atherosclerosis defined by single-cell RNA-sequencing and mass cytometry. Circ Res. (2018) 122:1675–88. 10.1161/CIRCRESAHA.117.312513 29545366 PMC5993603

[12] Rifkin IR Leadbetter EA Busconi L Viglianti G Marshak-Rothstein A. Toll-like receptors, endogenous ligands, and systemic autoimmune disease. Immunol Rev. (2005) 204:27–42. 10.1111/j.0105-2896.2005.00239.x 15790348

[13] Cole JE Kassiteridi C Monaco C. Toll-like receptors in atherosclerosis: a “Pandora's box” of advances and controversies. Trends Pharmacol Sci. (2013) 34:629–36. 10.1016/j.tips.2013.09.008 24139612

[14] Edfeldt K Swedenborg J Hansson GK Yan ZQ. Expression of toll-like receptors in human atherosclerotic lesions: a possible pathway for plaque activation. Circulation. (2002) 105:1158–61. 10.1161/circ.105.10.1158 11889007

[15] Sessa R. Infectious burden and atherosclerosis: a clinical issue. World J Clin Cases. (2014) 2:240–9. 10.12998/wjcc.v2.i7.240 25032197 PMC4097149

[16] Zimmer S Grebe A Latz E. Danger signaling in atherosclerosis. Circ Res. (2015) 116:323–40. 10.1161/CIRCRESAHA.116.301135 25593277

[17] Lanter BB Sauer K Davies DG. Bacteria present in carotid arterial plaques are found as biofilm deposits which may contribute to enhanced risk of plaque rupture. MBio. (2014) 5:e01206–14. 10.1128/mBio.01206-14 24917599 PMC4056553

[18] Gibson MS Domingues N Vieira OV. Lipid and non-lipid factors affecting macrophage dysfunction and inflammation in atherosclerosis. Front Physiol. (2018) 9:654. 10.3389/fphys.2018.00654 29997514 PMC6029489

[19] Brown MS Ho YK Goldstein JL. The cholesteryl ester cycle in macrophage foam cells. continual hydrolysis and re-esterification of cytoplasmic cholesteryl esters. J Biol Chem. (1980) 255:9344–52. 7410428

[20] Papac-Milicevic N Busch CJL Binder CJ. Malondialdehyde epitopes as targets of immunity and the implications for atherosclerosis. Adv Immunol. (2016) 131:1–59 10.1016/bs.ai.2016.02.001 27235680 PMC5892703

[21] Nahrendorf M Swirski FK. Abandoning M1/M2 for a network model of macrophage function. Circ Res. (2016) 119:414–7. 10.1161/CIRCRESAHA.116.309194 27458196 PMC4965179

[22] Robbins CS Hilgendorf I Weber GF Theurl I Iwamoto Y Figueiredo JL. Local proliferation dominates lesional macrophage accumulation in atherosclerosis. Nat Med. (2013) 19:1166–72. 10.1038/nm.3258 23933982 PMC3769444

[23] Murphy AJ Tall AR. Proliferating macrophages populate established atherosclerotic lesions. Circ Res. (2014) 114:236–8. 10.1161/CIRCRESAHA.113.302813 24436425 PMC4332765

[24] Christ A Günther P Lauterbach MAR Duewell P Biswas D Pelka K. Western diet triggers NLRP3-dependent innate immune reprogramming. Cell. (2018) 172:162–75.e14. 10.1016/j.cell.2017.12.013 29328911 PMC6324559

[25] Zysset D Weber B Rihs S Brasseit J Freigang S Riether C. TREM-1 links dyslipidemia to inflammation and lipid deposition in atherosclerosis. Nat Commun. (2016) 7:1–16. 10.1038/ncomms13151 27762264 PMC5080444

[26] Havel RJ Eder HA Bragdon JH. The distribution and chemical composition of ultracentrifugally separated lipoproteins in human serum. J Clin Invest. (1955) 34:1345–53. 10.1172/JCI103182 13252080 PMC438705

[27] Hodge SJ Hodge GL Holmes M Reynolds PN. Flow cytometric characterization of cell populations in bronchoalveolar lavage and bronchial brushings from patients with chronic obstructive pulmonary disease. Cytom Part B Clin Cytom. (2004) 6:27–34. 10.1002/cyto.b.20020 15351979

[28] Ni K O'Neill HC. Improved FACS analysis confirms generation of immature dendritic cells in long-term stromal-dependent spleen cultures. Immunol Cell Biol. (2000) 78:196–204. 10.1046/j.1440-1711.2000.00897.x 10849107

[29] Zi Y Yi-An Y Bing J Lee DS Kim J Wu T. Sirt6-induced autophagy restricted TREM-1-mediated pyroptosis in ox-LDL-treated endothelial cells: relevance to prognostication of patients with acute myocardial infarction. Cell Death Discov. (2020) 5:88. 10.1038/s41420-019-0168-4 30993014 PMC6461678

[30] Ohisson BG Englund MCO Karlsson ALK Knutsen E Erixon C Skribeck H. Oxidized low density lipoprotein inhibits lipopolysaccharide-induced binding of nuclear factor-kappaB to DNA and the subsequent expression of tumor necrosis factor-alpha and interleukin-1beta in macrophages. J Clin Invest. (1996) 98:78–89. 10.1172/JCI118780 8690807 PMC507403

[31] Walton KA Cole AL Yeh M Subbanagounder G Krutzik SR Modlin RL. Specific phospholipid oxidation products inhibit ligand activation of toll-like receptors 4 and 2. Arterioscler Thromb Vasc Biol. (2003) 23:1197–203. 10.1161/01.ATV.0000079340.80744.B8 12775576

[32] Stewart CR Stuart LM Wilkinson K van Gils JM Deng J Halle A. CD36 ligands promote sterile inflammation through assembly of a Toll-like receptor 4 and 6 heterodimer. Nat Immunol. (2010) 11:155–61. 10.1038/ni.1836 20037584 PMC2809046

[33] Habets KLL Van Puijvelde GHM Van Duivenvoorde LM Van Wanrooij EJA De Vos P Tervaert JWC. Vaccination using oxidized low-density lipoprotein-pulsed dendritic cells reduces atherosclerosis in LDL receptor-deficient mice. Cardiovasc Res. (2010) 85:622–30. 10.1093/cvr/cvp338 19819882

[34] Jongstra-Bilen J Zhang CX Wisnicki T Li MK White-Alfred S Ilaalagan R. Oxidized low-density lipoprotein loading of macrophages downregulates TLR-induced proinflammatory responses in a gene-specific and temporal manner through transcriptional control. J Immunol. (2017) 199:2149–57. 10.4049/jimmunol.1601363 28784845

[35] Sheedy FJ Grebe A Rayner KJ Kalantari P Ramkhelawon B Carpenter SB. CD36 coordinates NLRP3 inflammasome activation by facilitating intracellular nucleation of soluble ligands into particulate ligands in sterile inflammation. Nat Immunol. (2013) 14:812–20. 10.1038/ni.2639 23812099 PMC3720827

[36] Fong LG Fong TAT Cooper AD. (1991). Inhibition of lipopolysaccharide-induced interleukin-1beta mRNA expression in mouse macrophages by oxidized low density lipoprotein. J Lipid Res. 32:1899–1910. 1816321

[37] Hamilton JA Myers D Jessup W Cochrane F Byrne R Whitty G. Oxidized LDL can induce macrophage survival, DNA synthesis, and enhanced proliferative response to CSF-1 and GM-CSF. Arterioscler Thromb Vasc Biol. (1999) 19:98–105. 10.1161/01.ATV.19.1.98 9888871

[38] Perrin-Cocon L Coutant F Agaugué S Deforges S André P Lotteau V. (2001). Oxidized low-density lipoprotein promotes mature dendritic cell transition from differentiating monocyte. J. Immunol. 167:3785–91. 10.4049/jimmunol.167.7.3785 11564795

[39] Chung SV Kang BY Kim YK Pak YK Cho D Trinchieri G. (2000). Oxidized low density lipoprotein inhibits interleukin-12 production in lipopolysaccharide-activated mouse macrophages via direct interactions between peroxisome proliferator-activated receptor-gamma and nuclear factor-kappa B. J Biol Chem. 275:32681–7. 10.1074/jbc.M002577200 10934192

[40] Jiang Y Wang M Huang K Zhang Z Shao N Zhang Y. Oxidized low-density lipoprotein induces secretion of interleukin-1beta by macrophages via reactive oxygen species-dependent NLRP3 inflammasome activation. Biochem Biophys Res Commun. (2012) 425:121–6. 10.1016/j.bbrc.2012.07.011 22796220

[41] Liu W Yin Y Zhou Z He M Dai Y. OxLDL-induced IL-1beta secretion promoting foam cells formation was mainly via CD36 mediated ROS production leading to NLRP3 inflammasome activation. Inflamm Res. (2014) 63:33–43. 10.1007/s00011-013-0667-3 24121974

[42] Kannan Y Sundaram K Narasimhulu CA Parthasarathy S Wewers MD. Oxidatively modified low density lipoprotein (LDL) inhibits TLR2 and TLR4 cytokine responses in human monocytes but not in macrophages. J Biol Chem. (2012) 287:23479–88. 10.1074/jbc.M111.320960 22613713 PMC3390624

[43] Bekkering S Quintin J Joosten LAB Van Der Meer JWM Netea MG Riksen NP. Oxidized low-density lipoprotein induces long-term proinflammatory cytokine production and foam cell formation via epigenetic reprogramming of monocytes. Arterioscler Thromb Vasc Biol. (2014) 34:1731–8. 10.1161/ATVBAHA.114.303887 24903093

[44] Farhat K Riekenberg S Heine H Debarry J Lang R Mages J. Heterodimerization of TLR2 with TLR1 or TLR6 expands the ligand spectrum but does not lead to differential signaling. J Leukoc Biol. (2008) 83:692–701. 10.1189/jlb.0807586 18056480

[45] Hoebe K Georgel P Rutschmann S Du X Mudd S Crozat K. CD36 is a sensor of diacylglycerides. Nature. (2005) 433:523–7. 10.1038/nature03253 15690042

[46] Schröder NWJ Heine H Alexander C Manukyan M Eckert J Hamann L. Lipopolysaccharide binding protein binds to triacylated and diacylated lipopeptides and mediates innate immune responses. J Immunol. (2004) 173:2683–91. 10.4049/jimmunol.173.4.2683 15294986

[47] Triantafilou M Gamper FGJ Haston RM Mouratis MA Morath S Hartung T. Membrane sorting of toll-like receptor (TLR)-2/6 and TLR2/1 heterodimers at the cell surface determines heterotypic associations with CD36 and intracellular targeting. J Biol Chem. (2006) 281:31002–11. 10.1074/jbc.M602794200 16880211

[48] Manukyan M Triantafilou K Triantafilou M Mackie A Nilsen N Espevik T. Binding of lipopeptide to CD14 induces physical proximity of CD14, TLR2 and TLR1. Eur J Immunol. (2005) 35:911–21. 10.1002/eji.200425336 15714590

[49] Ranoa DRE Kelley SL Tapping RI. Human lipopolysaccharide-binding protein (LBP) and CD14 independently deliver triacylated lipoproteins to toll-like receptor 1 (TLR1) and TLR2 and enhance formation of the ternary signaling complex. J Biol Chem. (2013) 288:9729–41. 10.1074/jbc.M113.453266 23430250 PMC3617275

[50] Bzowska M Nogiec A Skrzeczynska-Moncznik J Mickowska B Guzik K Pryjma J. Oxidized LDLs inhibit TLR-induced IL-10 production by monocytes: a new aspect of pathogen-accelerated atherosclerosis. Inflammation. (2012) 35:1567–84. 10.1007/s10753-012-9472-3 22556042 PMC3397235

[51] Erridge C Kennedy S Spickett CM Webb DJ. Oxidized phospholipid inhibition of toll-like receptor (TLR) signaling is restricted to TLR2 and TLR4: roles for CD14, LPS-binding protein, and MD2 as targets for specificity of inhibition. J Biol Chem. (2008) 283:24748–59. 10.1074/jbc.M800352200 18559343 PMC3259833

[52] Von Schlieffen E Oskolkova OV Schabbauer G Gruber F Blüml S Genest M. Multi-hit inhibition of circulating and cell-associated components of the toll-like receptor 4 pathway by oxidized phospholipids. Arterioscler Thromb Vasc Biol. (2009) 29:356–62. 10.1161/ATVBAHA.108.173799 19112167

[53] Lopes-Virella MF Virella G Orchard TJ Koskinen S Evans RW Becker DJ. Antibodies to oxidized LDL and LDL-containing immune complexes as risk factors for coronary artery disease in diabetes mellitus. Clin Immunol. (1999) 90:165–72. 10.1006/clim.1998.4631 10080827

[54] Saad AF Virella G Chassereau C Boackle RJ Lopes-Virella MF. OxLDL immune complexes activate complement and induce cytokine production by MonoMac 6 cells and human macrophages. J Lipid Res. (2006) 47:1975–83. 10.1194/jlr.M600064-JLR200 16804192

[55] Rhoads JP Lukens JR Wilhelm AJ Moore JL Mendez-Fernandez Y Kanneganti T-D. Oxidized low-density lipoprotein immune complex priming of the Nlrp3 inflammasome involves TLR and FcγR cooperation and is dependent on CARD9. J Immunol. (2017) 198:2105–14. 10.4049/jimmunol.1601563 28130494 PMC5318843

[56] Shang L Daubeuf B Triantafilou M Olden R Dépis F Raby AC. Selective antibody intervention of toll-like receptor 4 activation through Fc γ receptor tethering. J Biol Chem. (2014) 289:15309–18. 10.1074/jbc.M113.537936 24737331 PMC4140888

[57] Prasad A Clopton P Ayers C Khera A De Lemos JA Witztum JL. Relationship of autoantibodies to MDA-LDL and ApoB-immune complexes to sex, ethnicity, subclinical atherosclerosis, and cardiovascular events. Arterioscler Thromb Vasc Biol. (2017) 37:1213–21. 10.1161/ATVBAHA.117.309101 28473443 PMC5500201

[58] Heit B Kim H Cosío G Castaño D Collins R Lowell CA. Multimolecular signaling complexes enable syk-mediated signaling of CD36 internalization. Dev Cell. (2013) 24:372–83. 10.1016/j.devcel.2013.01.007 23395392 PMC3586299

[59] Soto-Pantoja DR Kaur S Roberts DD. CD47 signaling pathways controlling cellular differentiation and responses to stress. Crit Rev Biochem Mol Biol. (2015) 50:212–30. 10.3109/10409238.2015.1014024 25708195 PMC4822708

[60] Nishi K Itabe H Uno M Kitazato KT Horiguchi H Shinno K. Oxidized LDL in carotid plaques and plasma associates with plaque instability. Arterioscler Thromb Vasc Biol. (2002) 22:1649–54. 10.1161/01.ATV.0000033829.14012.18 12377744

[61] Levitan I Volkov S Subbaiah PV. Oxidized LDL: diversity, patterns of recognition, and pathophysiology. Antioxid Redox Signal. (2010) 13:39–75. 10.1089/ars.2009.2733 19888833 PMC2877120

[62] Holvoet P Mertens A Verhamme P Bogaerts K Beyens G Verhaeghe R. Circulating oxidized LDL is a useful marker for identifying patients with coronary artery disease. Atheroscler Thromb Vasc Biol. (2001) 21:844–8. 10.1161/01.ATV.21.5.844 11348884

[63] Netea MG Nold-Petry CA Nold MF Joosten LAB Opitz B van Der Meer JHM. Differential requirement for the activation of the inflammasome for processing and release of IL-1beta in monocytes and macrophages. Blood. (2009) 113:2324–35. 10.1182/blood-2008-03-146720 19104081 PMC2652374

[64] Vromman A Ruvkun V Shvartz E Wojtkiewicz G Santos Masson G Tesmenitsky Y. Stage-dependent differential effects of interleukin-1 isoforms on experimental atherosclerosis. Eur Heart J. (2019) 40:2482–91. 10.1093/eurheartj/ehz008 30698710 PMC6685323

[65] Irmscher S Brix SR Zipfel SLH Halder LD Mutlutürk S Wulf S. Serum FHR1 binding to necrotic-type cells activates monocytic inflammasome and marks necrotic sites in vasculopathies. Nat Commun. (2019) 10:2961. 10.1038/s41467-019-10766-0 31273197 PMC6609651

[66] Pussinen PJ Paju S Koponen J Viikari JSA Taittonen L Laitinen T. Association of childhood oral infections with cardiovascular risk factors and subclinical atherosclerosis in adulthood. JAMA Netw Open. (2019) 2:e192523. 10.1001/jamanetworkopen.2019.2523 31026022 PMC6487573

[67] Tumurkhuu G Dagvadorj J Porritt RA Crother TR Shimada K Tarling EJ. Chlamydia pneumoniae hijacks a host autoregulatory IL-1β loop to drive foam cell formation and accelerate atherosclerosis. Cell Metab. (2018) 28:432–48.e4. 10.1016/j.cmet.2018.05.027 29937375 PMC6125162

[68] Almeida-da-Silva CLC Alpagot T Zhu Y Lee SS Roberts BP Hung SC. Chlamydia pneumoniae is present in the dental plaque of periodontitis patients and stimulates an inflammatory response in gingival epithelial cells. Microb Cell. (2019) 6:197–208. 10.15698/mic2019.04.674 30956972 PMC6444558

[69] Sokolova M Ranheim T Louwe MC Halvorsen B Yndestad A Aukrust P. NLRP3 inflammasome: a novel player in metabolically induced inflammation-potential influence on the myocardium. J Cardiovasc Pharmacol. (2019) 74:276–84. 10.1097/FJC.0000000000000704 31584530

[70] Karasawa T Takahashi M. The crystal-induced activation of NLRP3 inflammasomes in atherosclerosis. Inflamm Regen. (2017) 37:18. 10.1186/s41232-017-0050-9 29259717 PMC5725911

[71] Banerjee J Mishra N Damle G Dhas Y. Beyond LDL-c: The importance of serum oxidized LDL in predicting risk for type 2 diabetes in the middle-aged Asian Indians. Diabetes Metab Syndr. (2019) 13:206–13. 10.1016/j.dsx.2018.08.036 30641698

[72] Russo A Pirisinu I Vacca C Reginato E Tomaro ES Pippi R. An intensive lifestyle intervention reduces circulating oxidised low-density lipoprotein and increases human paraoxonase activity in obese subjects. Obes Res Clin Pract. (2018) 12:108–14. 10.1016/j.orcp.2016.11.006 27956218

[73] Karunakaran D Geoffrion M Wei L Gan W Richards L Shangari P. Targeting macrophage necroptosis for therapeutic and diagnostic interventions in atherosclerosis. Sci Adv. (2016) 2:e1600224. 10.1126/sciadv.1600224 27532042 PMC4985228

[74] Hsu S Koren E Chan Y Koscec M Sheehy A Kolodgie F. Effects of everolimus on macrophage-derived foam cell behavior. Cardiovasc Revascularization Med. (2014) 15:269–77. 10.1016/j.carrev.2014.05.007 24972512

[75] Kurdi A Roth L Van der Veken B Van Dam D De Deyn PP De Doncker M. Everolimus depletes plaque macrophages, abolishes intraplaque neovascularization and improves survival in mice with advanced atherosclerosis. Vascul Pharmacol. (2019) 113:70–6. 10.1016/j.vph.2018.12.004 30590134

[76] Cai Z He Y Chen Y. Role of mammalian target of rapamycin in atherosclerosis. Curr Mol Med. (2018) 18:216–32. 10.2174/1566524018666180926163917 30259816

[77] Guo FX Wu Q Li P Zheng L Ye S Dai XY. The role of the LncRNA-FA2H-2-MLKL pathway in atherosclerosis by regulation of autophagy flux and inflammation through mTOR-dependent signaling. Cell Death Differ. (2019) 26:1670–87. 10.1038/s41418-018-0235-z 30683918 PMC6748100

