# Supplementary material for: Phenotype and Response to PAMPs of Human Monocyte-Derived Foam Cells Obtained by Long-Term Culture in the Presence of oxLDLs
Source: Front Immunol. 2020 Aug 4;11:1592. doi: 10.3389/fimmu.2020.01592 (PMC7417357; doi:10.3389/fimmu.2020.01592)
Supplement: Supplementary file 1 [file Table_1.pdf]

Supplementary Table 1. Flow cytometry analysis of surface receptors expression in prolonged-hMDMs and hMDFCs presented as % of positive cells (Overtone subtraction). Data are the means ± SD of three to five independent experiments.

markers	% of positive cells (Overtone subtraction) Mean ± SD	
	**prolonged-hMDMs**	**prolonged-hMDFCs**
**TLR1**	44 ± 24	37 ± 17
**TLR2**	53 ± 15	50 ± 7
**TLR4**	50 ± 12	52 ± 16
**TLR6**	32 ± 10	26 ± 1
**CD11b**	56 ± 16	44 ± 16
**CD14**	55 ± 24	40 ± 14
**CD16**	75 ± 9	61 ± 8
**CD18**	93 ± 5	89 ± 8
**CD36**	65 ± 8	63 ± 18
**CD47**	92 ± 9	82 ± 19
**CD81**	89 ± 6	81 ± 10
**CD86**	84 ± 11	79 ± 16
**CD91**	83 ± 8	79 ± 5
