# Supplementary material for: Phenotype and Response to PAMPs of Human Monocyte-Derived Foam Cells Obtained by Long-Term Culture in the Presence of oxLDLs
Source: Front Immunol. 2020 Aug 4;11:1592. doi: 10.3389/fimmu.2020.01592 (PMC7417357; doi:10.3389/fimmu.2020.01592)
Supplement: Supplementary file 2 [file Table_2.pdf]

Supplementary Table 2. Flow cytometry analysis of surface receptors expression in prolonged-hMDMs and hMDFCs presented as MFI values. Data are the means ± SD of three to five independent experiments.

markers	Mean Fluorescence Intensity Mean ± SD	
	**prolonged-hMDMs**	**prolonged-hMDFCs**
**CD16**	1486 ± 626	1322 ± 570
**CD18**	8288 ± 2637	6929 ± 2549
**CD36**	3832 ± 1009	3964 ± 1485
**CD47**	3950 ± 291	2705 ± 533
**CD81**	4052 ± 1684	2943 ± 845
**CD86**	1926 ± 646	1880 ± 337
**CD91**	2370 ± 778	2201 ± 611
