# Supplementary material for: Phenotype and Response to PAMPs of Human Monocyte-Derived Foam Cells Obtained by Long-Term Culture in the Presence of oxLDLs
Source: Front Immunol. 2020 Aug 4;11:1592. doi: 10.3389/fimmu.2020.01592 (PMC7417357; doi:10.3389/fimmu.2020.01592)
Supplement: Supplementary file 3 [file Table_3.pdf]

SupplementaryTable 3. Concentration of cytokines (pg/mg) secreted by unstimulated and PAMPs-induced prolonged-hMDMs or prolonged-hMDFCs.*

Mean (pg/mg)** cytokine concentration ± SD***

	IL-6		IL-10		IL-12		IL-15		TNF		IL-1RA		IFN alpha		IL-2R	
	hMDMs	hMDFCs	hMDMs	hMDFCs	hMDMs	hMDFCs	hMDMs	hMDFCs	hMDMs	hMDFCs	hMDMs	hMDFCs	hMDMs	hMDFCs	hMDMs	hMDFCs
**control**	ND	ND	25 ± 25	17 ± 14	ND	ND	ND	ND	ND	ND	142 ± 78	281 ± 178	ND	ND	ND	ND
**st LPS**	1181 ± 167	809 ± 84	97 ± 68	52 ± 38	2049 ± 2211	326 ± 260	160 ± 7	ND	536 ± 261	267 ± 95	2302 ± 1550	1368 ± 569	103 ± 25	91 ± 34	357 ± 64	249 ± 165
**up LPS**	551 ± 380	304 ± 232	65 ± 56	31 ± 32	321 ± 222	81 ± 77	115 ± 61	ND	197 ± 131	45 ± 47	1116 ± 133	891 ± 130	101 ± 74	86 ± 48	292 ± 116	226 ± 175
**Pam2**	1666± 1271	1542 ± 820	128 ± 106	68 ± 63	1094 ± 1184	422 ± 290	ND	ND	394 ± 197	523 ± 246	1883 ± 724	1590 ± 432	147 ± 74	107 ± 53	367 ± 48	288 ± 122
**MALP-2**	977 ±727	773 ± 372	79 ± 68	54 ± 50	104 ± 42	126 ± 46	ND	ND	140 ± 94	132 ± 9	1072 ± 30	1133 ± 144	115 ± 88	103 ± 56	287 ± 117	237 ± 150
**LTA**	504 ± 347	106 ± 34	53 ± 42	21 ± 19	270 ± 131	19 ± 8	ND	ND	101 ± 79	25 ± 26	1092 ± 82	698 ± 23	103 ± 84	53 ± 32	260 ± 93	164 ± 132
**Pam3**	1176 ± 1360	504 ± 660	73 ± 71	33 ± 36	899 ± 1031	102 ± 73	ND	ND	361 ± 256	142 ± 126	2047 ± 1105	1072 ± 458	213 ± 210	46 ± 7	510 ± 363	317 ± 210
**CL075**	3696 ± 957	1610 ± 1433	272 ± 171	69 ± 73	22123 ± 19496	2670 ± 789	243 ± 142	ND	2401 ± 1361	1024 ± 942	8794 ± 2206	2956 ± 620	291 ± 176	122 ± 83	608 ± 273	370 ± 182
**Poly(I:C)**	51 ± 26	16 ± 17	46 ± 29	14 ± 6	55 ± 15	20 ± 13	295 ± 173	ND	334 ±305	48 ± 61	982 ± 303	733 ± 369	141 ± 45	99 ± 39	291 ± 50	151 ± 128
**PG**	1871 ± 1576	899 ± 873	82 ± 32	28 ± 14	608 ± 646	41 ± 14	ND	ND	1656 ± 1086	772 ± 577	1421 ± 487	881 ± 240	117 ± 75	38 ± 32	278 ± 74	185 ± 146

	MCP-1		MIP-1 alpha		MIP-1 beta		RANTES		IL-8		MIG		IP-10	
	hMDMs	hMDFCs	hMDMs	hMDFCs	hMDMs	hMDFCs	hMDMs	hMDFCs	hMDMs	hMDFCs	hMDMs	hMDFCs	hMDMs	hMDFCs
**control**	310 ± 69	1327 ± 541	105 ± 83	391 ± 355	47 ± 21	115 ± 96	ND	ND	33 ± 25	74 ± 18	ND	ND	ND	ND
**st LPS**	2598 ± 427	1688 ± 15	11540 ± 1804	9470 ± 2985	14097 ± 5318	9266 ± 3623	493 ± 179	188 ± 127	20276 ± 1500	17421 ± 4757	108 ± 71	ND	67 ± 20	12 ± 11
**up LPS**	2317 ± 1731	2292 ± 1246	6958 ± 5573	5046 ± 4552	6504 ± 2752	3571 ± 2275	167 ± 92	ND	13884 ± 9282	10239 ± 10139	45 ± 44	ND	38 ± 41	15 ± 20
**Pam2**	6942 ± 5792	3284 ± 2611	18195 ± 8112	17087 ± 12049	14227 ± 5557	13236 ± 5771	277 ± 233	271 ± 204	26751 ± 2682	18696 ± 6602	ND	ND	ND	ND
**MALP-2**	3819 ± 4005	2545 ± 1431	8603 ± 7845	10612 ± 6879	6872 ± 2812	7396 ± 499	98 ± 23	112 ± 66	15951 ± 12969	19067 ± 8064	ND	ND	ND	ND
**LTA**	2901 ± 2615	1320 ± 192	6589 ± 4433	3831 ± 3144	6087 ± 2114	2189 ± 608	119 ± 7	ND	12862 ± 8457	6518 ± 5678	ND	ND	ND	ND
**Pam3**	8841 ± 11303	3496 ± 4539	14206 ± 6999	8202 ± 5750	14702 ± 8766	7412 ± 4913	155 ± 260	41 ± 70	32139 ± 23791	16553 ± 15897	ND	ND	ND	ND
**CL075**	16226 ± 8965	7933 ± 5279	27404 ± 2019	19972 ± 4671	28584 ± 15289	13614 ± 3695	891 ± 695	133 ± 74	36084 ± 13703	23638 ± 7662	149 ± 8	50 ± 38	122 ± 72	54 ± 79
**Poly(I:C)**	4526 ± 716	3610 ± 1554	6444 ± 1928	3538 ± 4390	7750 ± 1090	2119 ± 1246	ND	ND	523 ± 321	375 ± 207	265 ± 90	53 ± 48	240 ± 39	37 ± 32
**PG**	2701 ± 1268	1633 ± 1493	5306 ± 2977	3092 ± 2532	1196 ± 1055	392 ± 300	ND	ND	26083 ± 5699	17646 ± 14657	ND	ND	ND	ND

ND - not detected

* Culture supernatants were collected 24h after stimulation.

** Results of multiplex cytokine analysis (expressed in pg/mL) were normalized to protein concentrations in corresponding cultures and expressed in picograms per milligram of total protein.

*** Data represent means and standard deviations of at least three independent experiments (each in triplicate).
