# Supplementary material for: Phenotype and Response to PAMPs of Human Monocyte-Derived Foam Cells Obtained by Long-Term Culture in the Presence of oxLDLs
Source: Front Immunol. 2020 Aug 4;11:1592. doi: 10.3389/fimmu.2020.01592 (PMC7417357; doi:10.3389/fimmu.2020.01592)
Supplement: Supplementary file 4 [file Image_1.pdf]

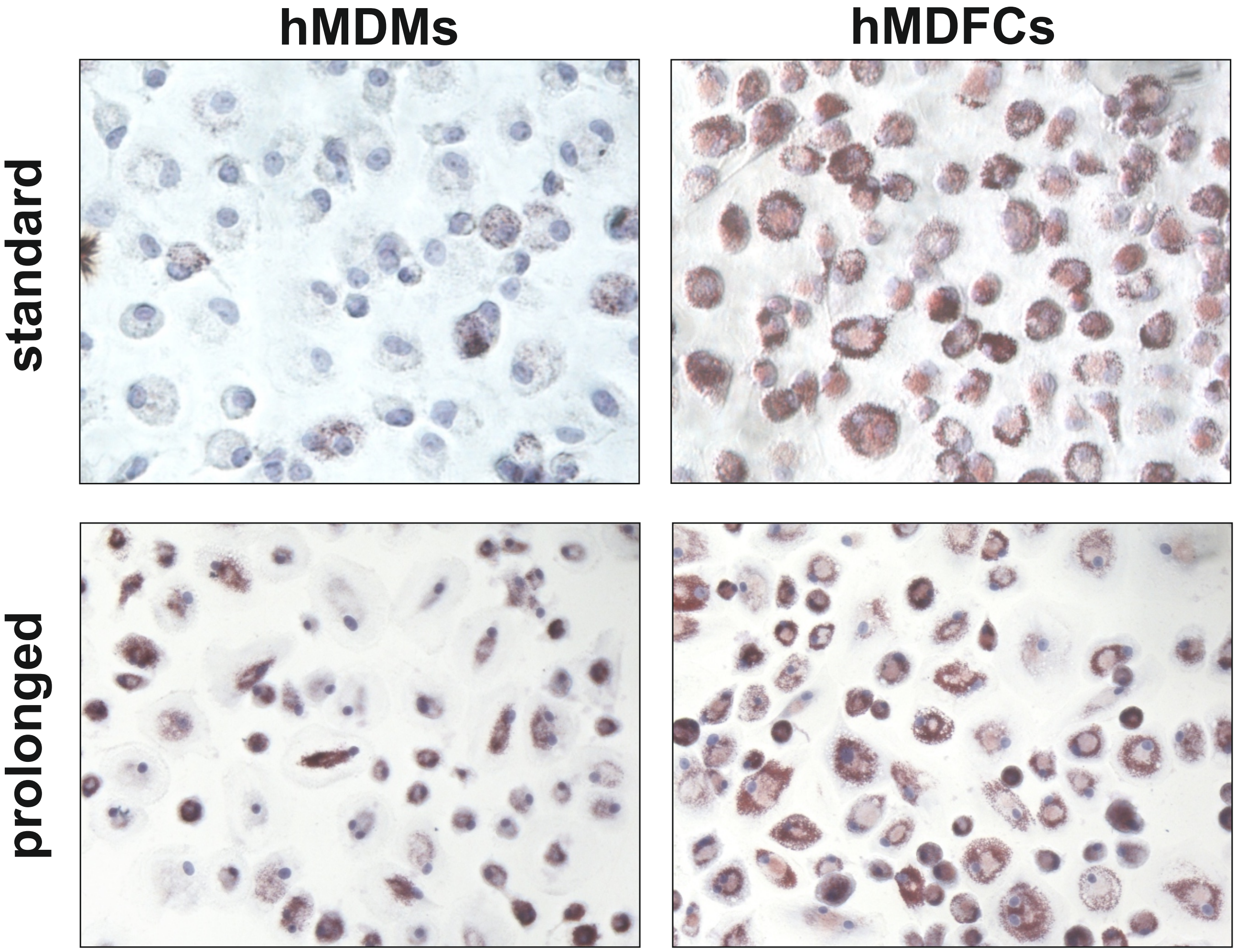

Supplementary Figure 1. Comparison of lipid-loading by hMDFCs obtained by standard and prolonged cell culture.
Standard/prolonged-hMDMs/hMDFCs were obtained as indicated in Materials and Methods, and Figure 1. The extent of cellular lipid accumulation was assessed by Oil Red O staining. The cells were counterstained with hematoxylin. Representative staining images are shown (magnification, x20).
