# Supplementary material for: Phenotype and Response to PAMPs of Human Monocyte-Derived Foam Cells Obtained by Long-Term Culture in the Presence of oxLDLs
Source: Front Immunol. 2020 Aug 4;11:1592. doi: 10.3389/fimmu.2020.01592 (PMC7417357; doi:10.3389/fimmu.2020.01592)
Supplement: Supplementary file 5 [file Image_2.pdf]

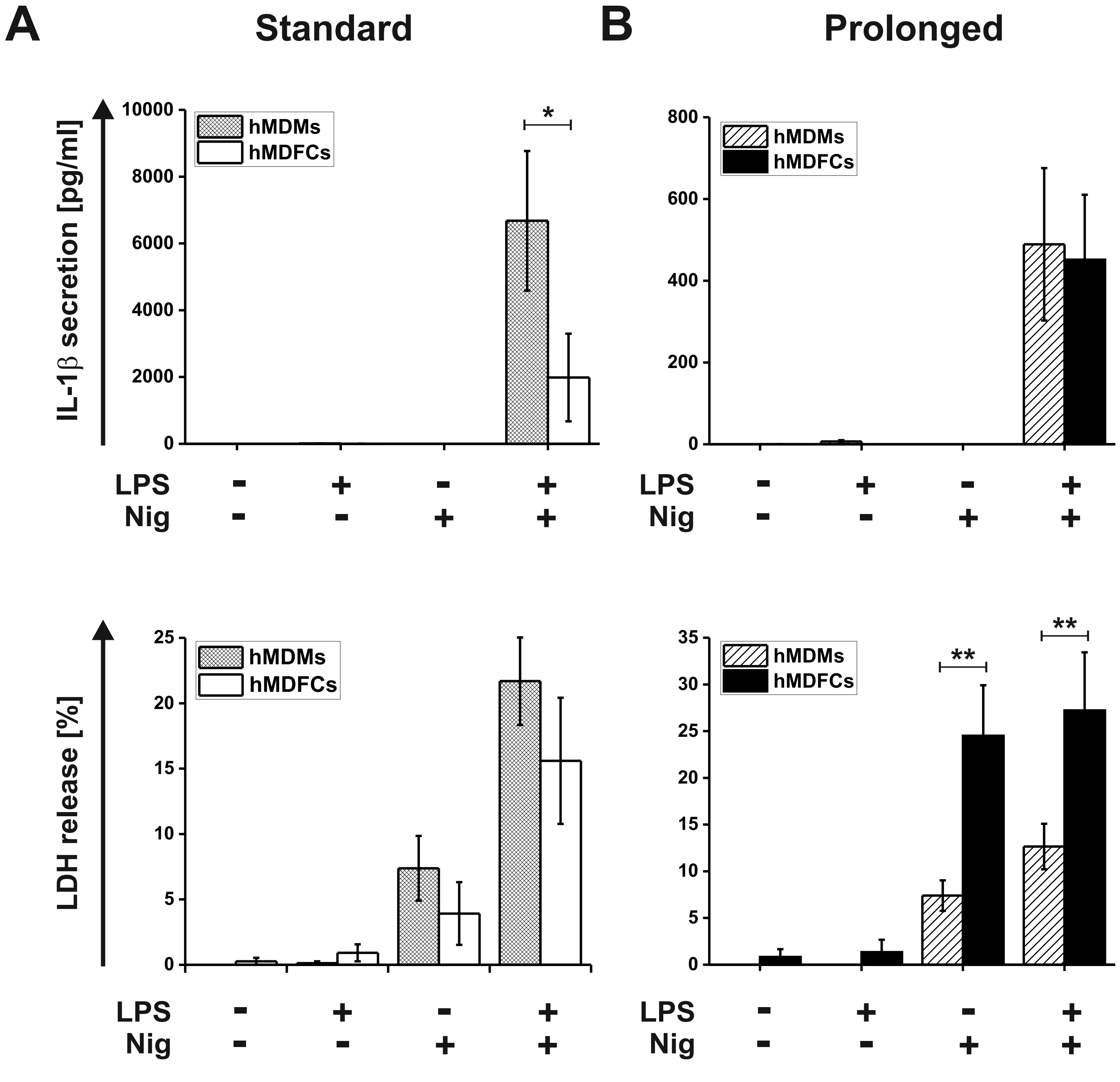

Supplementary Figure 2. Pyroptosis and IL-1β secretion by (A) standard- and (B) prolonged-hMDMs and hMDFCs.
Standard/prolonged-hMDMs/hMDFCs were obtained as indicated in Materials and Methods, and Figure 1. The cells were then primed with stLPS (1µg/mL) for 4 hours and stimulated with nigericin (10µM) for next 20 hours. Levels of IL-1β and LDH activity in supernatants were determined by Human IL-1β ELISA Set II, and Pierce™ LDH Cytotoxicity Assay Kit, respectively. LDH release was normalized to total LDH content. Bars and error bars represent means ± SEM from seven independent experiments (each in duplicate, n=14). *, $p<0.05$,**, $p<0.01$.
