# Supplementary material for: Phenotype and Response to PAMPs of Human Monocyte-Derived Foam Cells Obtained by Long-Term Culture in the Presence of oxLDLs
Source: Front Immunol. 2020 Aug 4;11:1592. doi: 10.3389/fimmu.2020.01592 (PMC7417357; doi:10.3389/fimmu.2020.01592)
Supplement: Supplementary file 6 [file Image_3.pdf]

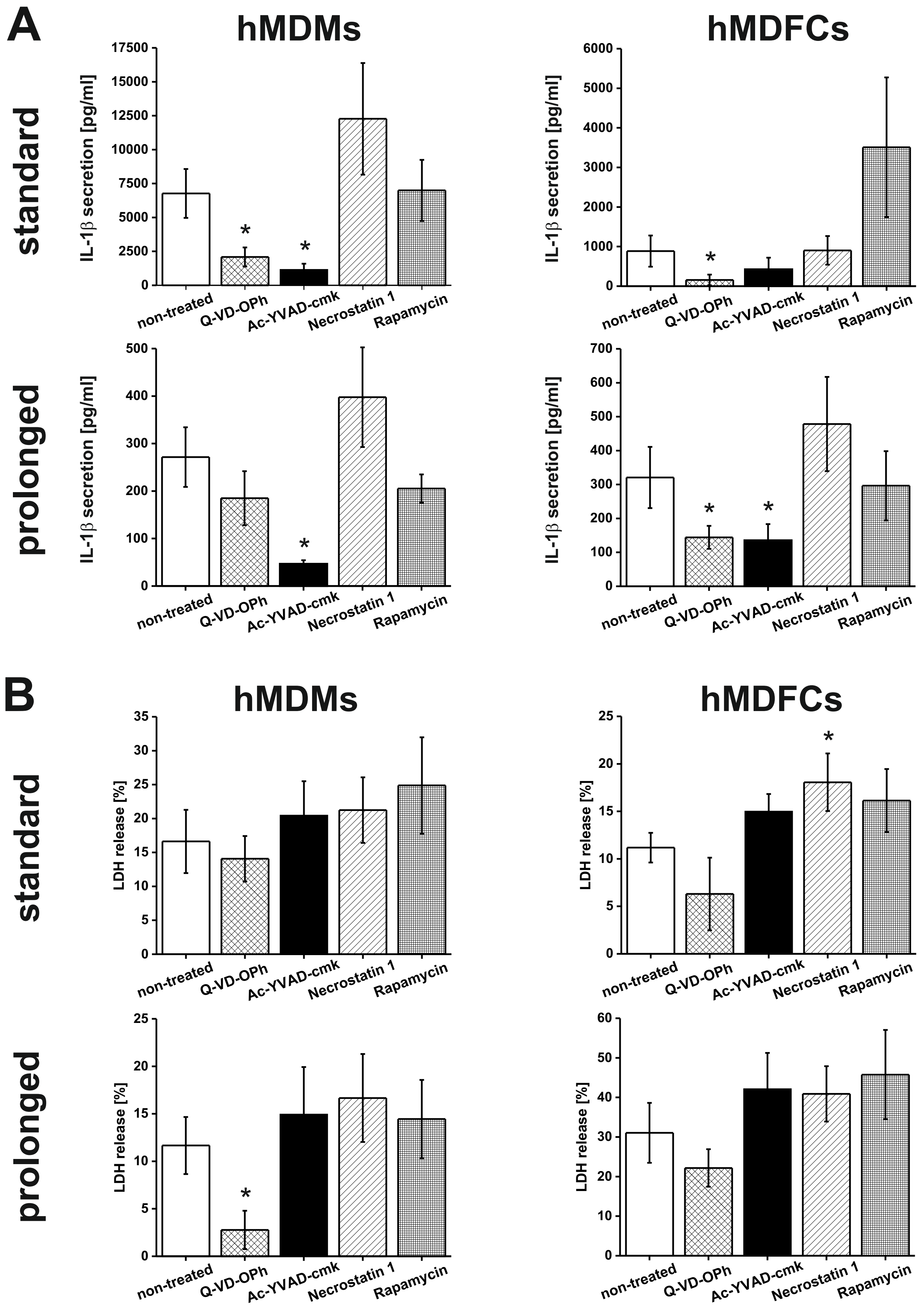

Supplementary Figure 3. The effect of caspases, RIP1 kinase and mTOR inhibition on IL-1β secretion (A) and pyroptosis (B) in standard- and prolonged-hMDFCs.
Standard/prolonged-hMDMs/hMDFCs were obtained as indicated in Materials and Methods, and Figure 1. The cells were pretreated for 1h with 20 μM Q-VD-OPh, 50 μM Ac-YVAD-cmk, 20 μM necrostatin-1 or 50nM rapamycin (inhibitor of caspase-3, -1, -8, -9; caspase-1; RIP1 kinase or mTOR, respectively) and then primed with 1 μg/mL LPS for 4h. Next, the cells were treated with 10 μM nigericin for next 20h. **(A)** IL-1β concentrations in supernatants were determined using Human IL-1β ELISA Set II. **(B)** LDH release was measured using Pierce™ LDH Cytotoxicity Assay Kit. LDH release was normalized to total LDH content. Bars and error bars represent means ± SEM from four (Q-VD-OPh) or five (others) independent experiments (each in duplicate, n>= 8).*, $p<0.05$ versus non-treated cells.
